# Supplementary material for: Dynamic multicolor emissions of multimodal phosphors by Mn2+ trace doping in self-activated CaGa4O7
Source: Nat Commun. 2024 Apr 13;15:3209. doi: 10.1038/s41467-024-47431-0 (PMC11016074; doi:10.1038/s41467-024-47431-0)
Supplement: Supplementary file 1 — Supplementary Information [file 41467_2024_47431_MOESM1_ESM.docx]

**Supplementary Information**

**Dynamic multicolor emissions of multimodal phosphors by Mn^2+^ trace doping in self-activated CaGa_4_O_7_**

Yiqian Tang^1,2,6^, Yiyu Cai^1,2,6^, Kunpeng Dou^1,2^, Jianqing Chang^1,2^, Wei Li^1,2^, Shanshan Wang^1,2^, Mingzi Sun^3^, Bolong Huang^3,^*, Xiaofeng Liu^4^, Jianrong Qiu^4^, Lei Zhou^5^, Mingmei Wu^5^, Jun-Cheng Zhang^1,^^2,^*

^1^College of Physics and Optoelectronic Engineering, Faculty of Information Science and Engineering, Ocean University of China, Qingdao 266100, China

^2^Engineering Research Center of Advanced Marine Physical Instruments and Equipment of Education Ministry of China, and Key Laboratory of Optics and Optoelectronics of Qingdao, Ocean University of China, Qingdao 266100, China

^3^Department of Applied Biology and Chemical Technology, The Hong Kong Polytechnic University, Hong Kong SAR, China

^4^College of Optical Science and Engineering, State Key Laboratory of Modern Optical Instrumentation, Zhejiang University, Hangzhou 310027, China

^5^School of Chemical Engineering and Technology, Sun Yat-sen University, Zhuhai 519082, China

^6^These authors contributed equally: Yiqian Tang, Yiyu Cai.

*Email: bhuang@polyu.edu.hk (B.H.); [zhangjuncheng@ouc.edu.cn](mailto:zhangjuncheng@ouc.edu.cn) (J.-C.Z.)

Contents

Supplementary Tables 1−4

**Supplementary Note 1.** **Controlled incorporation of Mn^2+^ ions and microstructural characterization (Supplementary Figures 1−5)**

**Supplementary Note 2. Dynamic multicolor PL and adjustable emission color-changing rates (Supplementary Figures 6−13)**

**Supplementary Note 3. Temperature-dependent multicolor reversal and thermally boosted Mn^2+^ emission (Supplementary Figures 14−18)**

Supplementary Note 4. Studies of defect states (Supplementary Figures 19−24)

**Supplementary Note 5. Physics and interactions in dynamic multimodal luminescence (Supplementary Figures 25−29)**

Supplementary Note 6. Applications in dynamic color and pattern displays under photo-thermo-mechanical stimulations (Supplementary Figures 30−37)

Supplementary Movies 1−3

Supplementary references

Supplementary Tables 1−4

**Supplementary Table 1 | Highlights of the dynamic multimodal luminescence of CaGa_4_O_7_:Mn^2+^ developed in this work, compared with other reported luminescent materials with related characteristics.**

|  | Photoexcitation time-dependent multicolor evolution | Temporal memory effect | Temperature-  dependent multicolor emission | Thermally enhanced emission | Durable  elastic ML | Suppl. Ref. |
| --- | --- | --- | --- | --- | --- | --- |
| MgGa_2_O_4_:Bi^3+^ | **√** | − | × | **√** | × | 1 |
| NaGdF_4_:Yb/Ce/Ho | **√** | − | **√** | **√** | × | 2 |
| MgGa_2_O_4_ | **√** | − | **√** | **√** | × | 3 |
| ImBr | **√** | − | × | × | × | 4 |
| CaZnGe_2_O_6_:Mn^2+^ | **√** | − | × | × | × | 5 |
| CaGa_4_O_7_:Mn^2+^ | **√** | **√** | **√** | **√** | **√** | This work |

**Supplementary Table 2 | Rietveld refinement parameters for as-synthesized Ca_1_**_−_***_x_*Ga_4_O_7_:*x*Mn^2+^ (*x* = 0−10×10**^−^**^4^) phosphors.**

| CaGa_4_O_7_:Mn^2+^ | *x* = 0 | 0.2×10^−4^ | 1×10^−4^ | 2×10^−4^ | 3×10^−4^ | 4×10^−4^ | 6×10^−4^ | 10×10^−4^ |
| --- | --- | --- | --- | --- | --- | --- | --- | --- |
| *T* | 298 K | | | | | | | |
| Space group | C2/c | | | | | | | |
| *a* (Å) | 13.127 | 13.124 | 13.122 | 13.121 | 13.120 | 13.120 | 13.119 | 13.118 |
| *b* (Å) | 9.118 | 9.116 | 9.115 | 9.115 | 9.115 | 9.115 | 9.114 | 9.112 |
| *c* (Å) | 5.612 | 5.611 | 5.610 | 5.610 | 5.610 | 5.610 | 5.609 | 5.608 |
| *V* (Å^3^) | 648.640 | 648.250 | 648.027 | 647.931 | 647.903 | 647.867 | 647.751 | 647.429 |
| *Z* | 4 | | | | | | | |
| *R*_wp_ (%) | 3.44 | 2.80 | 2.90 | 3.11 | 3.12 | 2.96 | 3.03 | 3.76 |
| *R*_p_ (%) | 2.55 | 2.14 | 2.18 | 2.34 | 2.38 | 2.21 | 2.24 | 2.81 |
| *χ*^2^ | 3.35 | 2.31 | 2.52 | 2.81 | 2.92 | 2.60 | 2.75 | 4.00 |

**Supplementary Table 3 | Structural parameters obtained from Rietveld refinement of XRD profiles for CaGa_4_O_7_ and CaGa_4_O_7_:Mn^2+^.**

| Material | Atom | Coordinates | | | 100×*U*_iso_ (Å^2^) |
| --- | --- | --- | --- | --- | --- |
|  |  | *x* | *y* | *z* |  |
| CaGa_4_O_7_ | Ca | 0.0000 | 0.2940 | 0.2500 | 2.608 |
|  | Ga1 | 0.1208 | 0.0623 | 0.7426 | 1.921 |
|  | Ga2 | 0.3394 | 0.0856 | 0.1854 | 1.981 |
|  | O1 | 0.1900 | 0.0750 | 0.0866 | 1.378 |
|  | O2 | 0.3898 | 0.0750 | -0.0929 | 1.540 |
|  | O3 | 0.1119 | 0.2596 | -0.3732 | 1.155 |
|  | O4 | 0.0000 | 0.0512 | 0.2500 | 2.193 |
| CaGa_4_O_7_:Mn^2+^ | Ca | 0.0000 | 0.2940 | 0.2500 | 2.541 |
|  | Ga1 | 0.1207 | 0.0626 | 0.7418 | 1.684 |
|  | Ga2 | 0.3393 | 0.0862 | 0.1859 | 1.758 |
|  | O1 | 0.1917 | 0.0740 | 0.0852 | 1.155 |
|  | O2 | 0.3909 | 0.0760 | -0.0947 | 1.458 |
|  | O3 | 0.1116 | 0.2593 | -0.3732 | 0.841 |
|  | O4 | 0.0000 | 0.0494 | 0.2500 | 2.112 |

**Supplementary Table 4 | Impurities in as-synthesized CaGa_4_O_7_ material measured by inductively coupled plasma-mass spectrometry (ICP-MS).**

| Element | Content (μg g^−1^) | Element | Content (μg g^−1^) | Element | Content (μg g^−1^) |
| --- | --- | --- | --- | --- | --- |
| Li | 0.011 | Se | 0.14 | Gd | 0.036 |
| Be | 0.0012 | Rb | 0.03 | Tb | 0.12 |
| B | 41.19 | Sr | 1.62 | Dy | 0.017 |
| Na | 13.99 | Y | 0.0006 | Ho | 0.054 |
| Mg | 0.77 | Zr | 0.012 | Er | 0.19 |
| Al | 11.71 | Nb | 0.081 | Tm | 0.022 |
| Si | 17.45 | Mo | 0.092 | Yb | 0.0016 |
| P | 0.36 | Ru | 0.11 | Lu | 0.0098 |
| S | 0.16 | Rh | 0.04 | Hf | 0.06 |
| K | 22.63 | Pd | 0.23 | Ta | 0.042 |
| Sc | 0.002 | Ag | 0.022 | W | 0.11 |
| Ti | 0.045 | Cd | 0.011 | Re | 0.054 |
| V | 0.12 | Sn | 0.05 | Os | 0.006 |
| Cr | 0.09 | Sb | 0.03 | Ir | 0.19 |
| Mn | 2.77 | Te | 0.15 | Pt | 0.18 |
| Fe | 2.54 | Ba | 0.017 | Au | 0.091 |
| Co | 0.26 | La | 0.15 | Hg | 0.182 |
| Ni | 0.13 | Ce | 0.022 | Tl | 0.13 |
| Cu | 13.25 | Pr | 0.1 | Pb | 0.036 |
| Zn | 3.54 | Nd | 0.08 | Bi | 0.15 |
| Ge | 1.55 | Sm | 0.0024 | Th | 0.13 |
| As | 0.08 | Eu | 0.0024 | U | 0.018 |

Supplementary Note 1. Controlled incorporation of Mn^2+^ ions and microstructural characterization


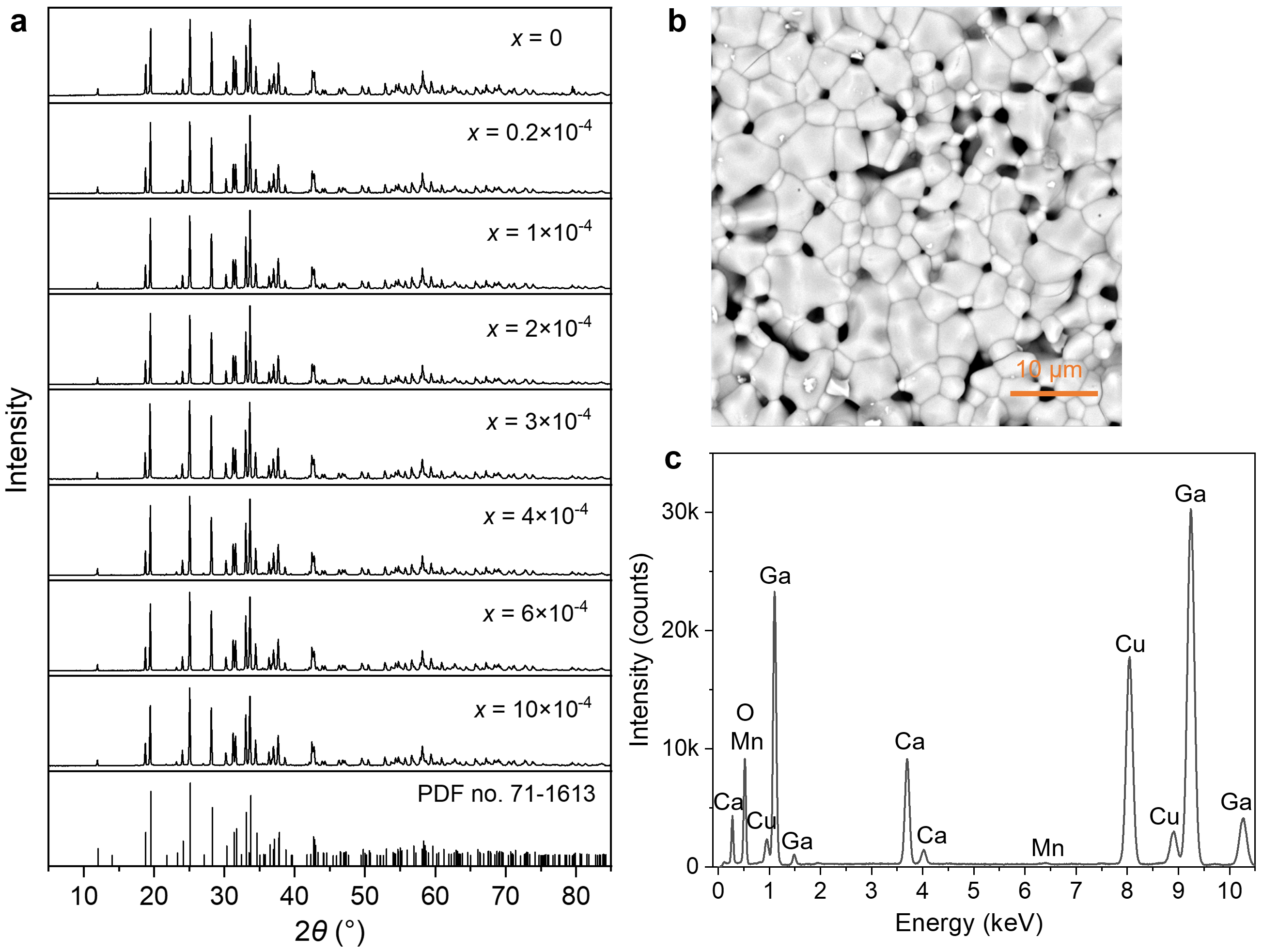


Supplementary Figure 1 | Characterization of the structure, morphology and elements of CaGa_4_O_7_:Mn^2+^. a, Powder XRD patterns of Ca_1-_*_x_*Ga_4_O_7_:*x*Mn^2+^ (*x* = 0, 0.2×10^−4^, 1×10^−4^, 2×10^−4^, 3×10^−4^, 4×10^−4^, 6×10^−4^, and 10×10^−4^), demonstrating the synthesized CaGa_4_O_7_:Mn^2+^ series as a single phase. The powder diffraction file (PDF) is no. 71-1613. b,c, SEM image and TEM-EDS spectrum of the synthesized Ca_1−_*_x_*Ga_4_O_7_:*x*Mn^2+^ (*x* = 1×10^−4^) pellet. The Cu element in the TEM-EDS spectrum arises from the copper mesh used in the TEM test.


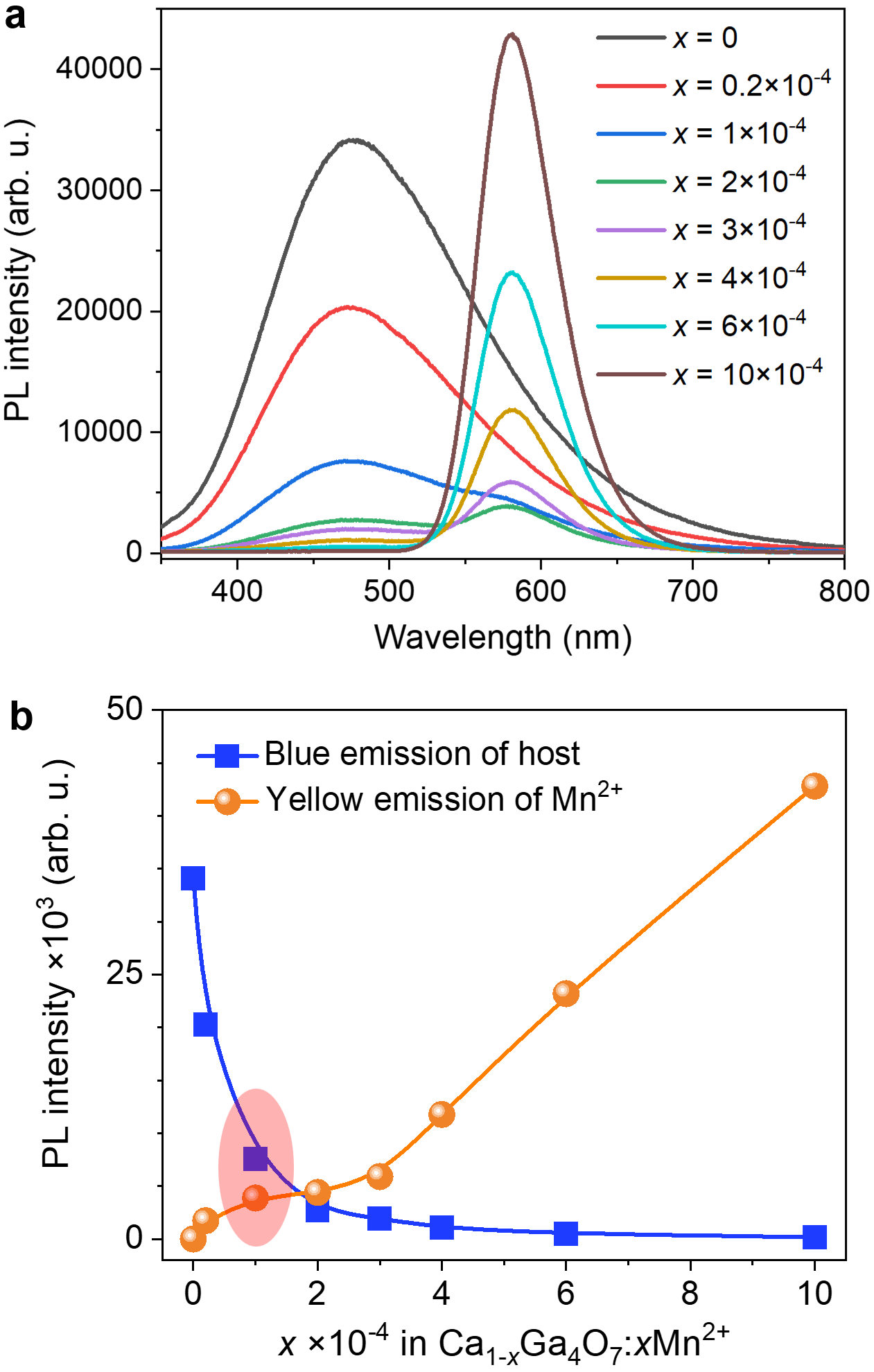


Supplementary Figure 2 | Screening of Mn^2+^ concentration based on the PL spectra of Ca_1−_*_x_*Ga_4_O_7_:*x*Mn^2+^. a, Steady-state PL spectra (*λ*_ex_ = 254 nm). b, The extracted blue- and yellow-emission intensity as a function of Mn^2+^ concentration. The red ellipse highlights the Mn^2+^ concentration of *x* = 1×10^−4^, which was the focus of this study for achieving dynamic multicolor evolution.


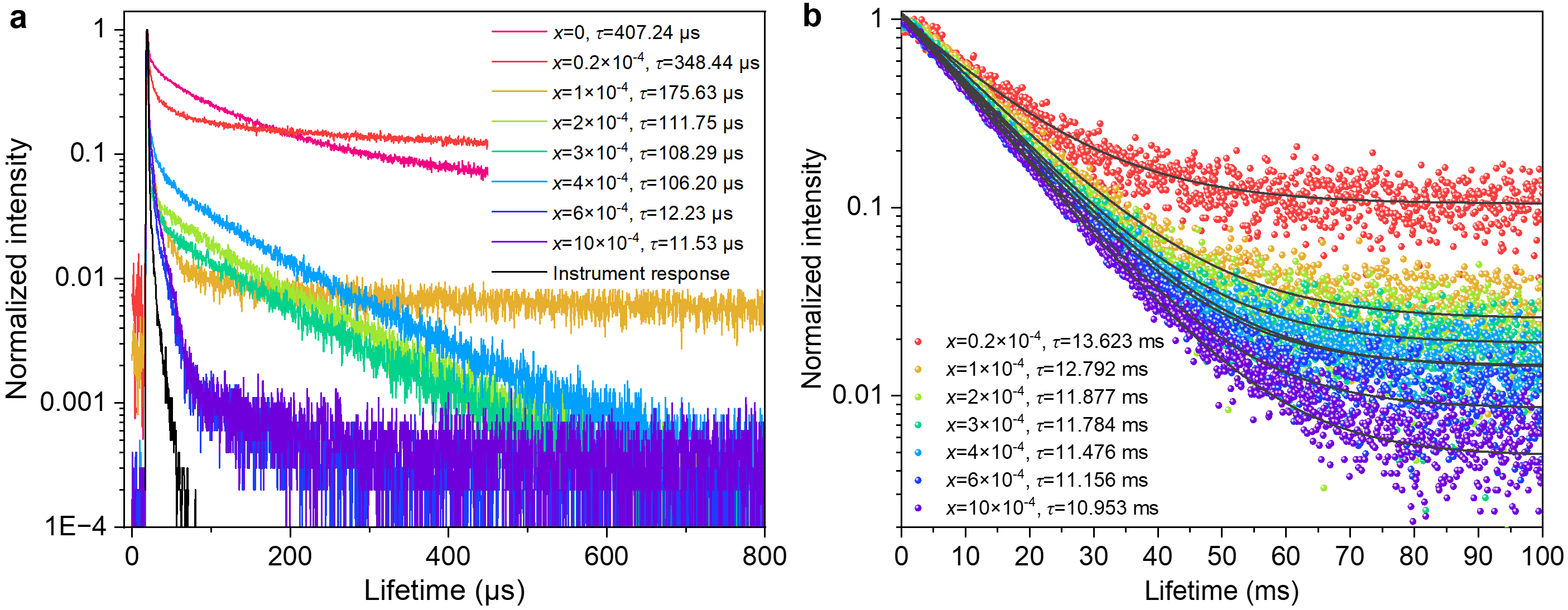


**Supplementary Figure 3 | PL decay curves of the blue and yellow emissions of the Ca_1_**_−_***_x_*Ga_4_O_7_:*x*Mn^2+^ (*x* = 0−10×10**^−^**^4^) phosphors at room temperature. a,** Blue emission (*λ*_ex_ = 260 nm, *λ*_em_ = 474 nm). **b,** Yellow emission (*λ*_ex_ = 254 nm, *λ*_em_ = 579 nm). The decay curves of blue emission were fitted by a bi-exponential function. Two lifetimes were obtained by fitting each curve, the first being the response time of the instrument (here a microsecond lamp was used as the excitation source) and the second being the lifetime of the tested sample, as shown in Supplementary Figure 3a. The decay curves of yellow emission were fitted by a monoexponential function. These results show that both the lifetime of blue emission and the lifetime of yellow emission are gradually shortened with increasing Mn^2+^ doping concentration. The former shortening may be due to the energy competition of the doped Mn^2+^ ions,^6^ while the latter shortening is possibly caused by the intensified non-radiative transitions of Mn^2+^ ions with the increase of Mn^2+^ doping concentration.^7^ It is noteworthy that no blue emission lifetime has been reported in CaGa_4_O_7_. As a comparison, the magnitude of the decay time of host emission in CaGa_4_O_7_ is much longer than the reported values (1.8 μs and 2.511 μs) for self-activated MgGa_2_O_4_ phosphor, which also has blue emission from host.^8,9^ The magnitude of the decay times of Mn^2+^ emission is similar to the previously reported values (10.927−7.083 ms) in Ca_1-_*_x_*Ga_4_O_7_:*x*Mn^2+^ (*x* = 0.002−0.1),^10^ suggesting that the luminescence of Mn^2+^ in CaGa_4_O_7_ is a spin-forbidden transition.


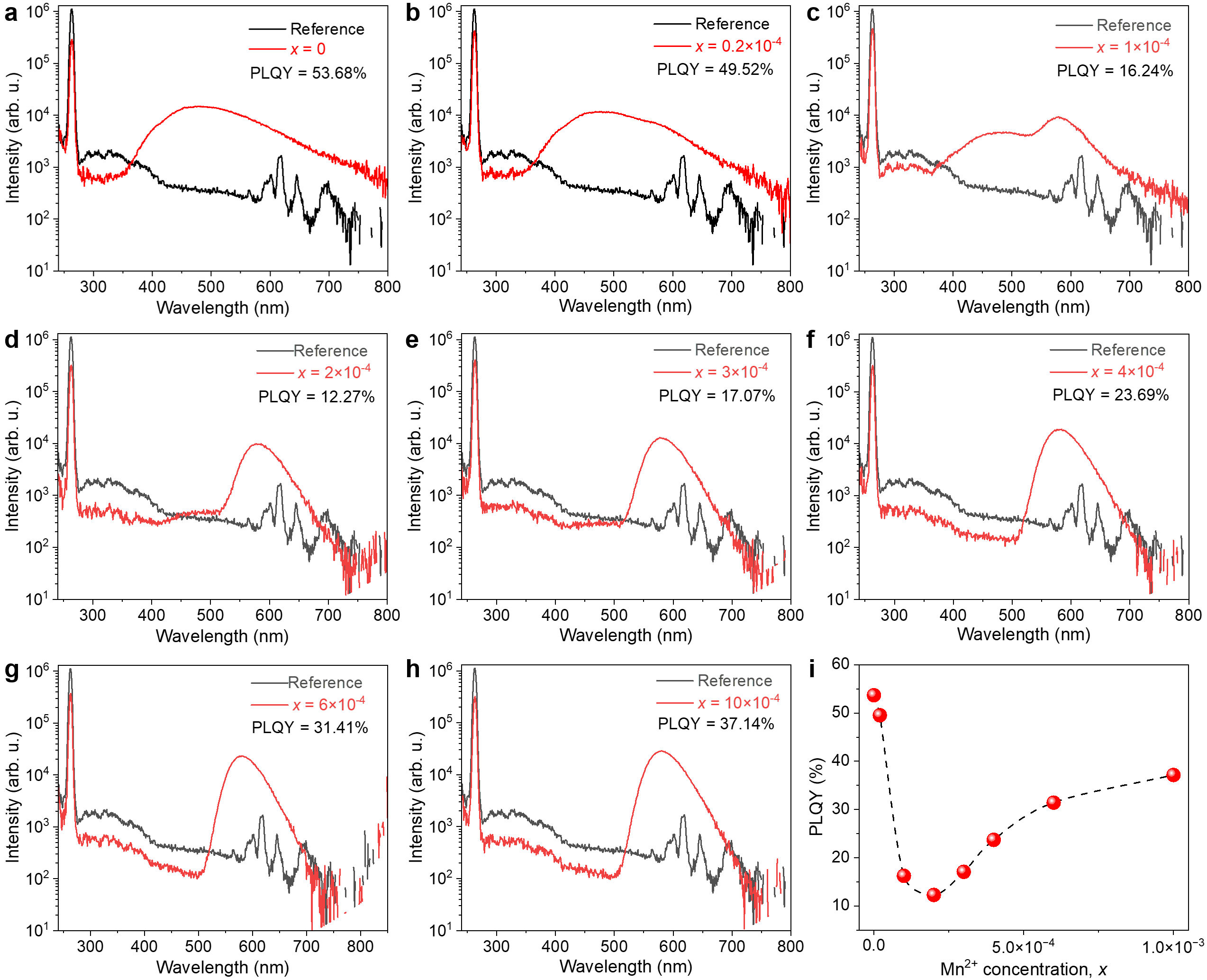


**Supplementary Figure 4 | PLQY of the CaGa_4_O_7_:Mn^2+^ phosphors at room temperature. a**−**h,** Absolute PLQY measurement of the Ca_1−_*_x_*Ga_4_O_7_:*x*Mn^2+^ (*x* = 0−10×10^−4^) phosphors. **i,** Mn^2+^ concentration-dependent PLQY of Ca_1−_*_x_*Ga_4_O_7_:*x*Mn^2+^. We noticed that the PLQY gradually decreased with increasing Mn^2+^ concentration from *x* = 0 to *x* = 2 × 10^−4^, while it gradually enhanced from *x* = 2 × 10^−4^ to *x* = 10 × 10^−4^. This is because the blue light emission of the CaGa_4_O_7_ host is burst by Mn^2+^ doping in the former stage, while the Mn^2+^ emission is continuously enhanced in the latter stage.


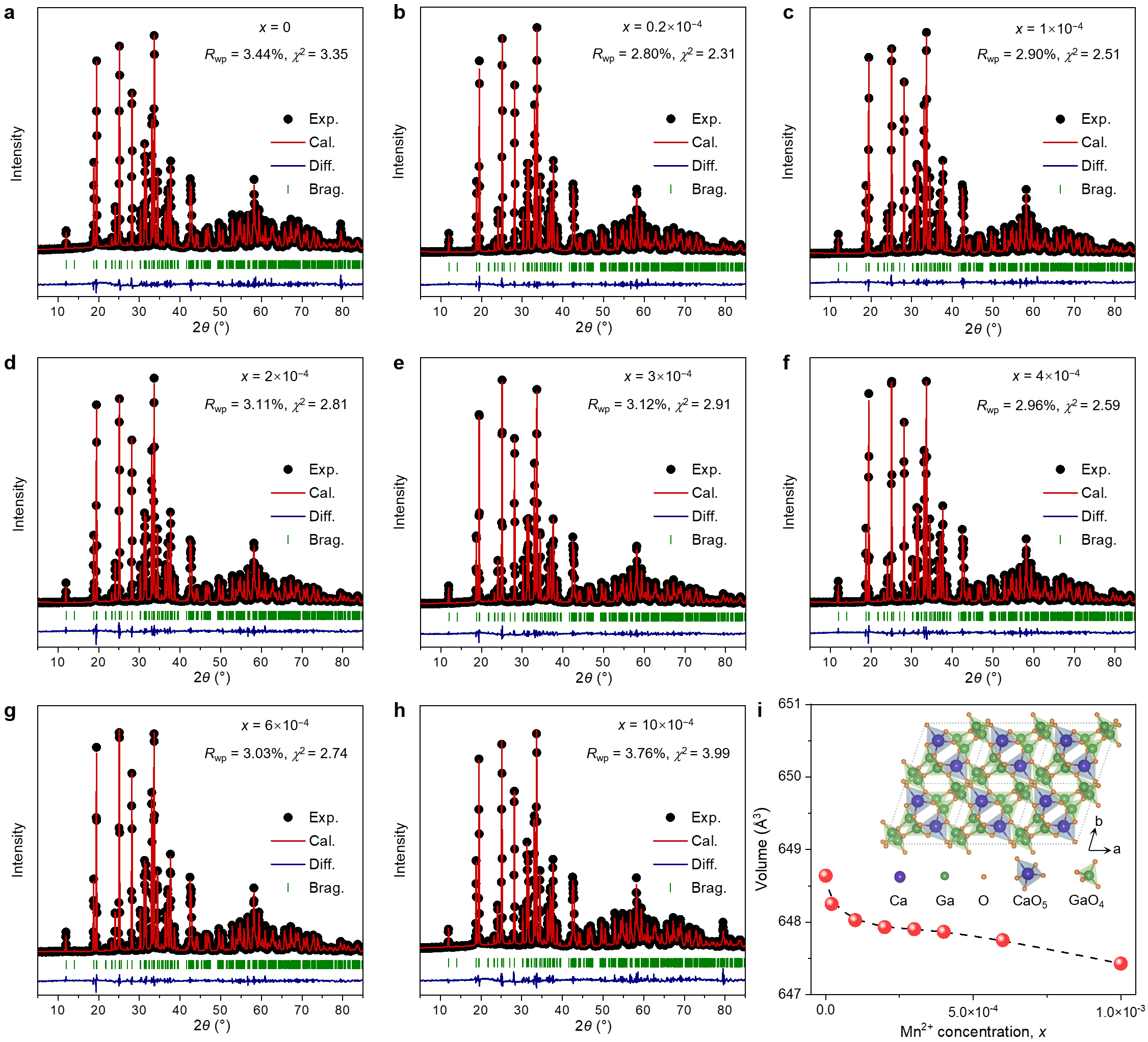


**Supplementary Figure 5 | Rietveld refined XRD profiles and calculated parameters of CaGa_4_O_7_:Mn^2+^. a−h,** Rietveld refinement of XRD patterns for Ca_1−_*_x_*Ga_4_O_7_:*x*Mn^2+^. The calculated crystallographic structural parameters are summarized in Supplementary Table 2. **i,** Refined volume of the unit cell for Ca_1−_*_x_*Ga_4_O_7_:*x*Mn^2+^. The inset displays the crystal structure of CaGa_4_O_7_. The monoclinic C2/c (No. 15) space group is adopted by CaGa_4_O_7_, and the crystal structure comprises two inequivalent Ga^3+^ sites and one Ca^2+^ site.^11^ Ca^2+^ is bonded to five O^2−^ atoms to form CaO_5_ trigonal bipyramids that share corners with ten GaO_4_ tetrahedra.

In theory, Mn^2+^ dopants are expected to occupy the Ca^2+^ sites due to the close radii and the crystal field effect. The radius percentage difference (Dr) values between Mn^2+^ (0.83 Å, coordination number (CN) = 6) and Ca^2+^ (1 Å, CN = 6) and between Mn^2+^ (0.66 Å, CN = 4) and Ga^3+^ (0.47 Å, CN = 4) are 17% and 40%, respectively.^12,13^ The calculation results indicate that the Mn^2+^ dopants are prone to occupy the Ca^2+^ sites because an acceptable Dr value cannot exceed 30%. Note that since there is no available radius data for Ca^2+^ with CN = 5, data of CN = 6 for Ca^2+^ and Mn^2+^ are used as reasonable approximations. Rietveld refinement analysis revealed a gradual decrease in the volume of the unit cell as the Mn^2+^ doping concentration increased, confirming the successful substitution of smaller Mn^2+^ ions for the Ca^2+^ sites. Moreover, Mn^2+^ in a five-coordinated environment emits yellow light,^14^ while Mn^2+^ in a tetra-coordinated environment emits green light instead of yellow light,^15^ which further confirms that Mn^2+^ dopants occupy the five-coordinated Ca^2+^ sites.

Supplementary Note 2. Dynamic multicolor PL and adjustable emission color-changing rates


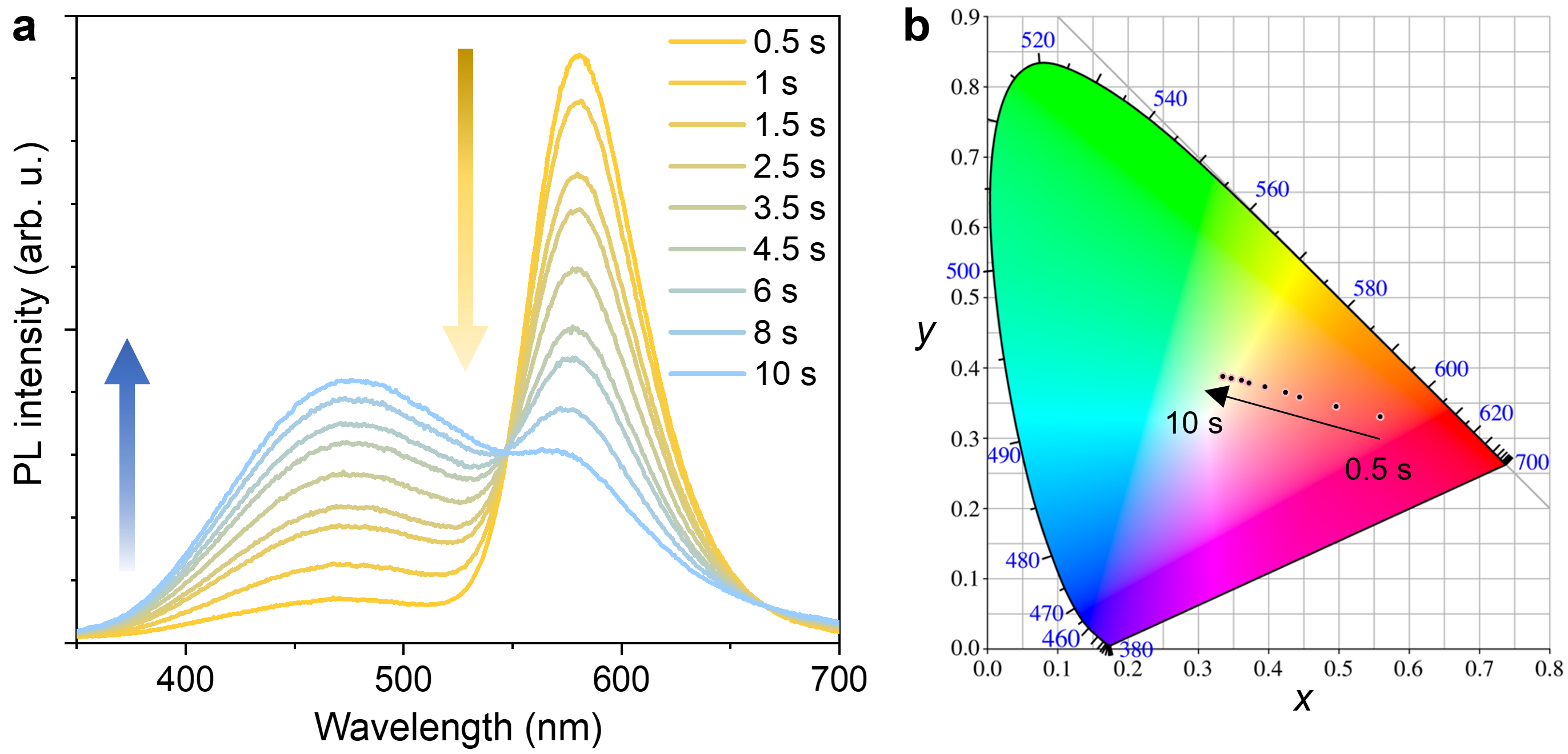


Supplementary Figure 6 | Photoexcitation time-dependent multicolor emission of CaGa_4_O_7_:Mn^2+^. a, Time-resolved PL spectra upon photoexcitation for different time (0.5−10 s). It shows that the intensity of the blue emission increases, while the intensity of the yellow emission decreases with the increase of photoexcitation time. Notably, their peak positions remain the same, that is, the peak position of the blue emission is at 474 nm and the peak position of the yellow emission is at 579 nm. b, Dependence of the CIE chromaticity coordinates on photoexcitation time (0.5−10 s).


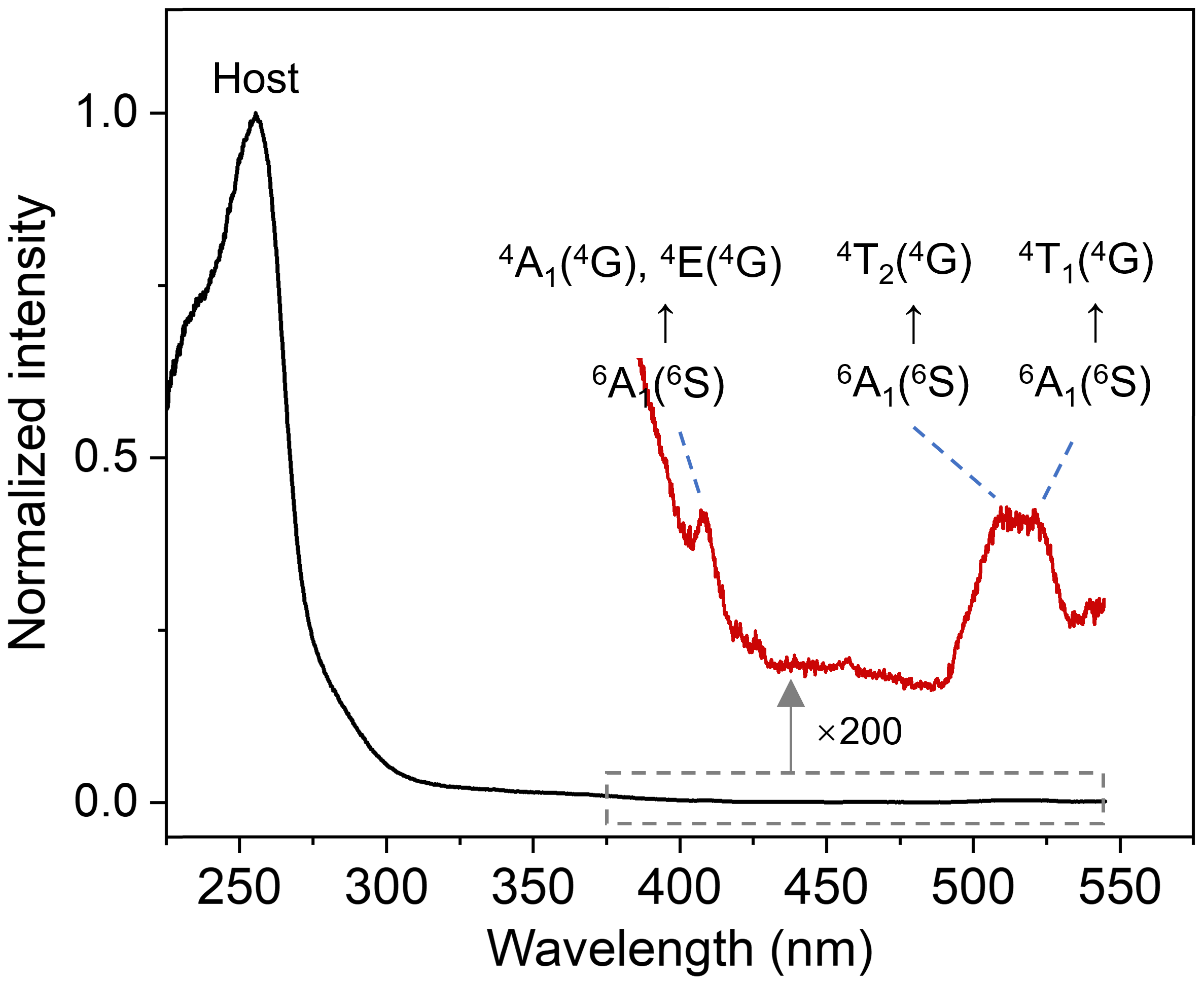


Supplementary Figure 7 | PL excitation spectrum of Ca_1−_*_x_*Ga_4_O_7_:*x*Mn^2+^ with *x* = 1×10^−4^ (*λ*_em_ = 579 nm). The enlarged spectrum (375−550 nm) shows the transitions of Mn^2+^ ions from ground state ^6^A_1_(^6^S) to excited states ^4^A_1_(^4^G), ^4^E(^4^G) (409 nm), ^4^T_2_(^4^G) (510 nm) and ^4^T_1_(^4^G) (522 nm).


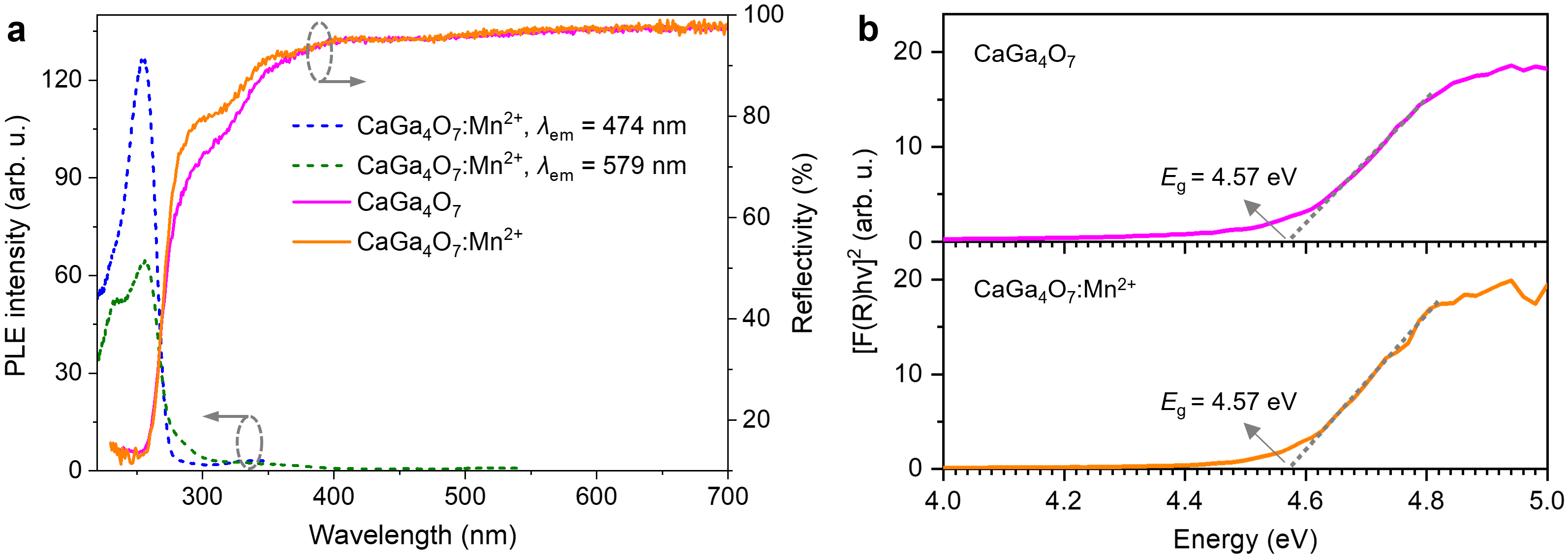


Supplementary Figure 8 | Optical bandgaps of CaGa_4_O_7_ and CaGa_4_O_7_:Mn^2+^. a, PL excitation spectra of CaGa_4_O_7_:Mn^2+^ (*λ*_em_ = 474 and 579 nm) and diffuse reflectance spectra of CaGa_4_O_7_ and CaGa_4_O_7_:Mn^2+^. The results show that the excitation band is correlated to the absorption band of CaGa_4_O_7_ and CaGa_4_O_7_:Mn^2+^ materials, indicating that the excitation of host and Mn^2+^ emission in CaGa_4_O_7_:Mn^2+^ is through the band-to-band transition. b, Curves of [F(R)hν]^2^ versus hν derived from the diffuse reflectance data for CaGa_4_O_7_ and CaGa_4_O_7_:Mn^2+^. The band gaps of both CaGa_4_O_7_ and CaGa_4_O_7_:Mn^2+^ were calculated to be 4.57 eV using the Kubelka−Munk function and the Tauc relation,^16,17^ indicating that microscale Mn^2+^ doping has no effect on the bandgap value of CaGa_4_O_7_.


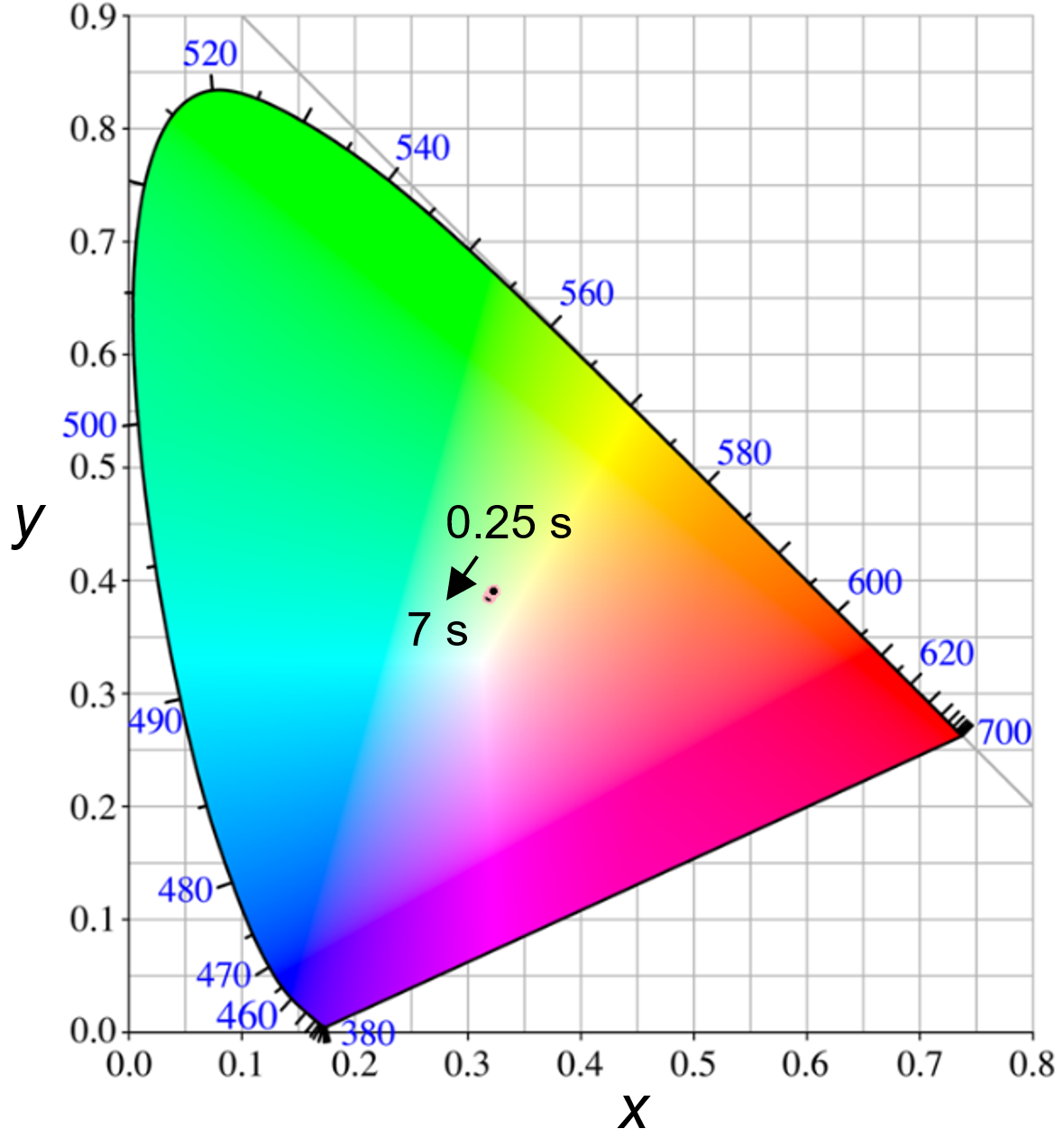


Supplementary Figure 9 | CIE chromaticity coordinates of the host emission from undoped CaGa_4_O_7_ with increasing photoexcitation time (0.25−7 s). The results show only minor changes in the CIE coordinates.


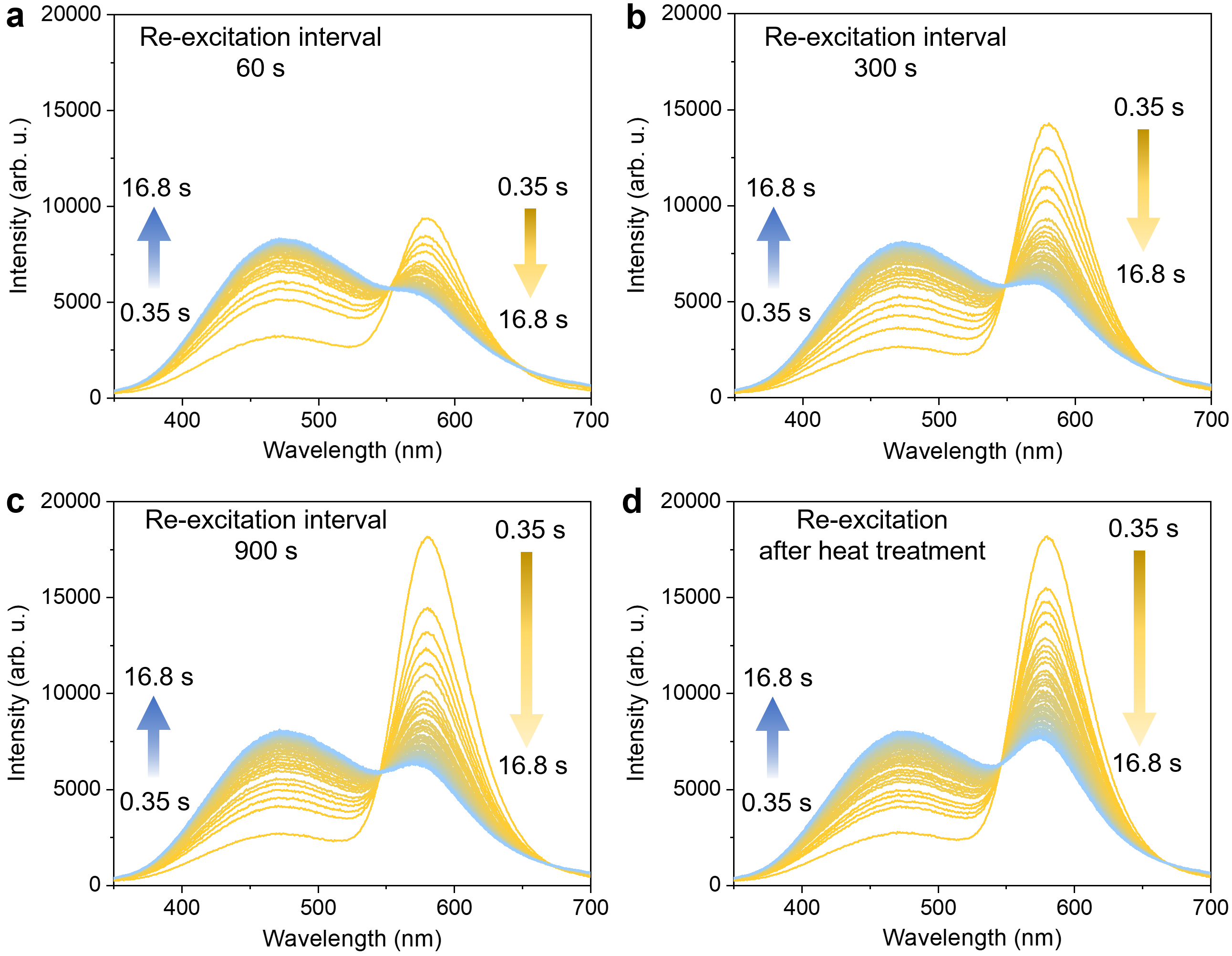


Supplementary Figure 10 | Time-resolved PL spectra of CaGa_4_O_7_:Mn^2+^ under re-excitation at different time intervals and after heat treatment. a−d, After 60 s, after 300 s, and after 900 s, and after heat treatment (473 K for 10 s). Each sample was pre-excited by a 254 nm UV lamp for 20 s prior to re-excitation.


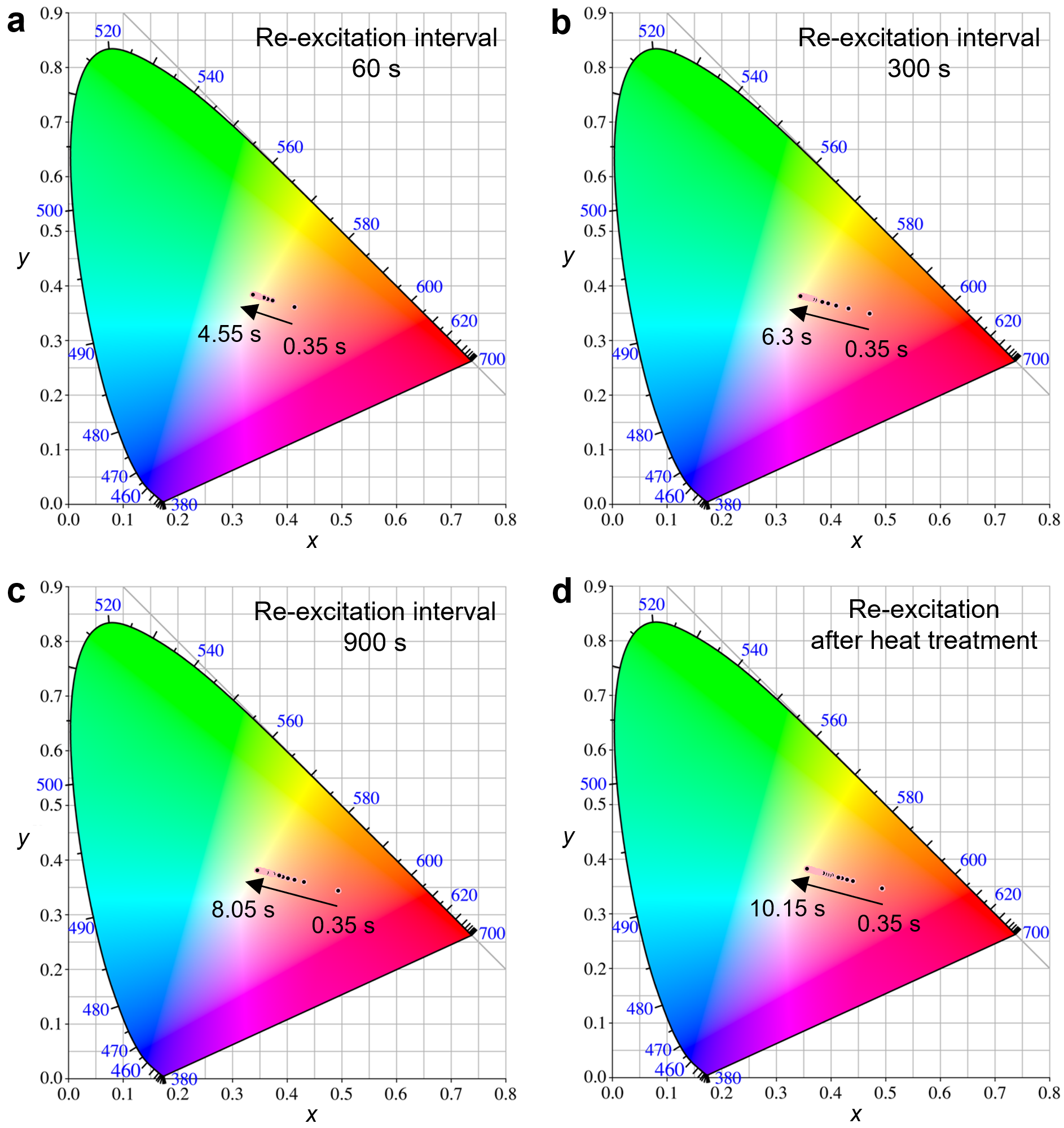


**Supplementary Figure 11 | Dependence of emission color-changing rates on re-excitation intervals and heat treatment. a,** Re-excitation interval of 60 s. **b,** Re-excitation interval of 300 s. **c,** Re-excitation interval of 900 s. **d,** Heat treatment (473 K, 10 s). These results illustrate that a shorter re-excitation interval leads to a faster evolution of the emission color, and a simple heat treatment can rapidly restore the evolutionary rate to the initial state when the phosphor was first photoexcited.


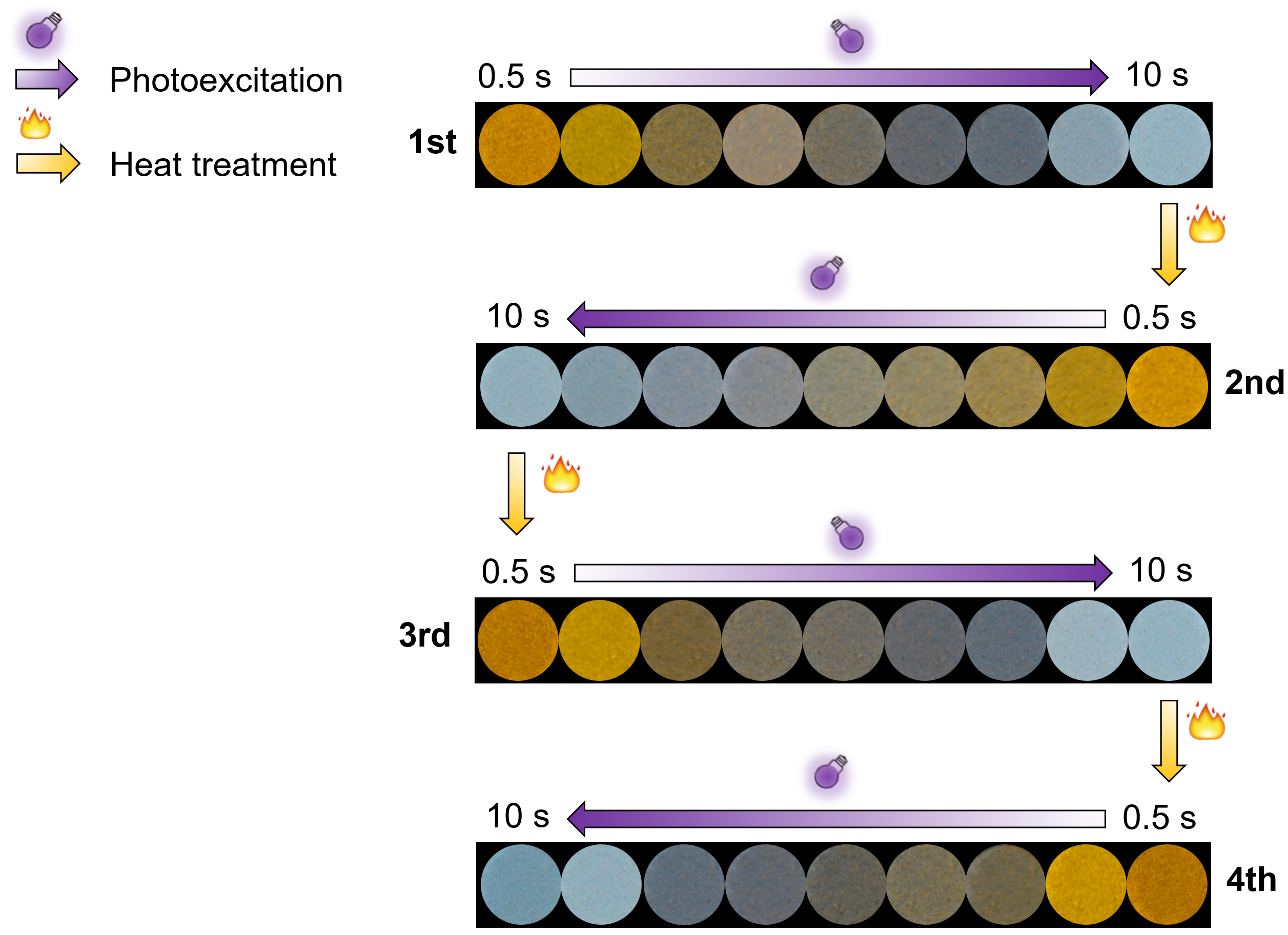


Supplementary Figure 12 | Multicolor evolution of PL from CaGa_4_O_7_:Mn^2+^ under cyclic photoexcitation and heat treatment. The photographs show the reproducible time-dependent multicolor emission by cycling the sample between photoexcitation (254 nm, 10 s) and heat treatment (473 K, 10 s).


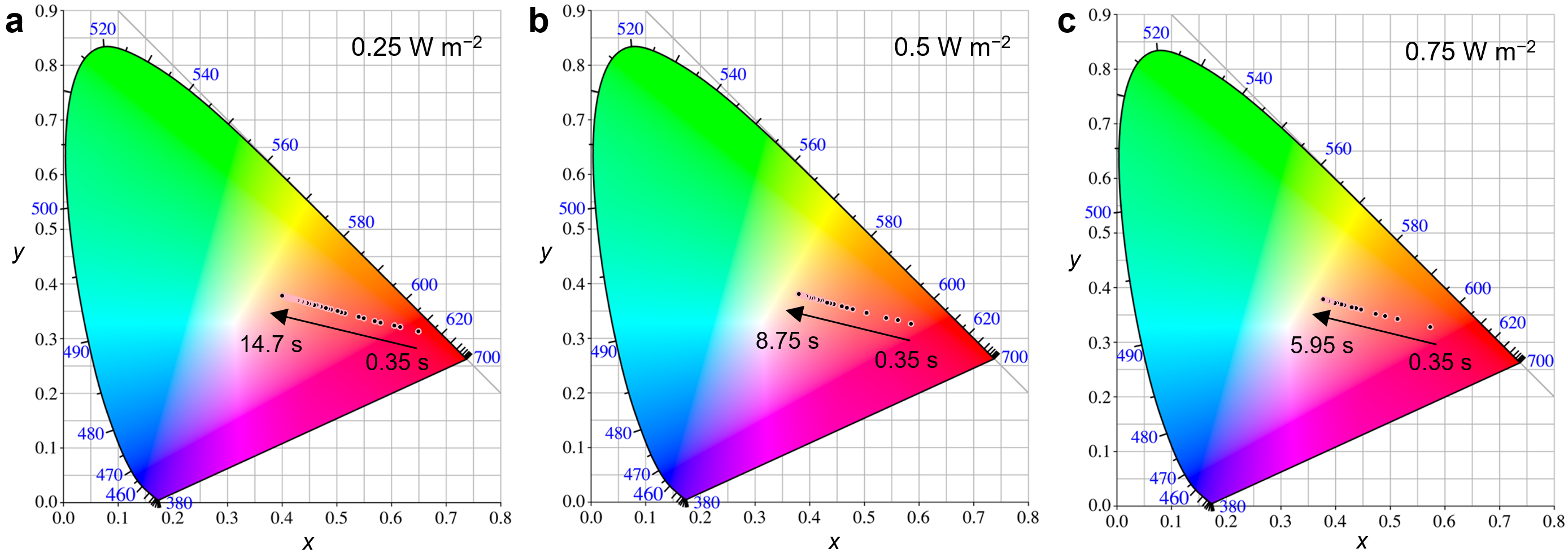


Supplementary Figure 13 | Dependence of emission color-changing rates on the optical power density. a−c, Standard CIE chromaticity graphs under different optical power densities (0.25, 0.5 and 0.75 W cm^−2^, *λ*_ex_ = 254 nm). These results illustrate that a higher optical power density leads to a faster evolution of the emission color.

Supplementary Note 3. Temperature-dependent multicolor reversal and thermally boosted Mn^2+^ emission


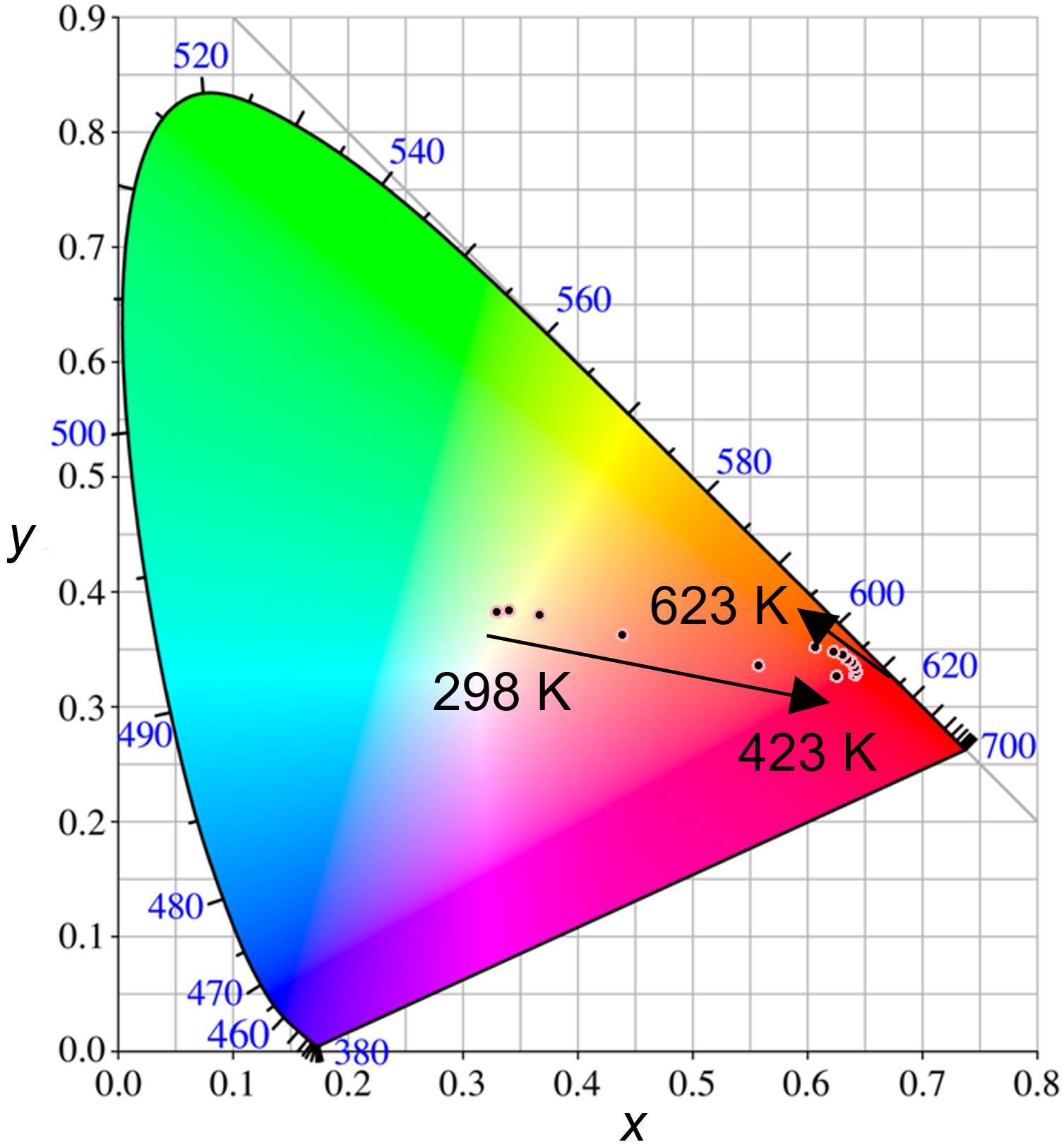


Supplementary Figure 14 | Dependence of the CIE chromaticity coordinates of the PL spectra of CaGa_4_O_7_:Mn^2+^ on temperature.


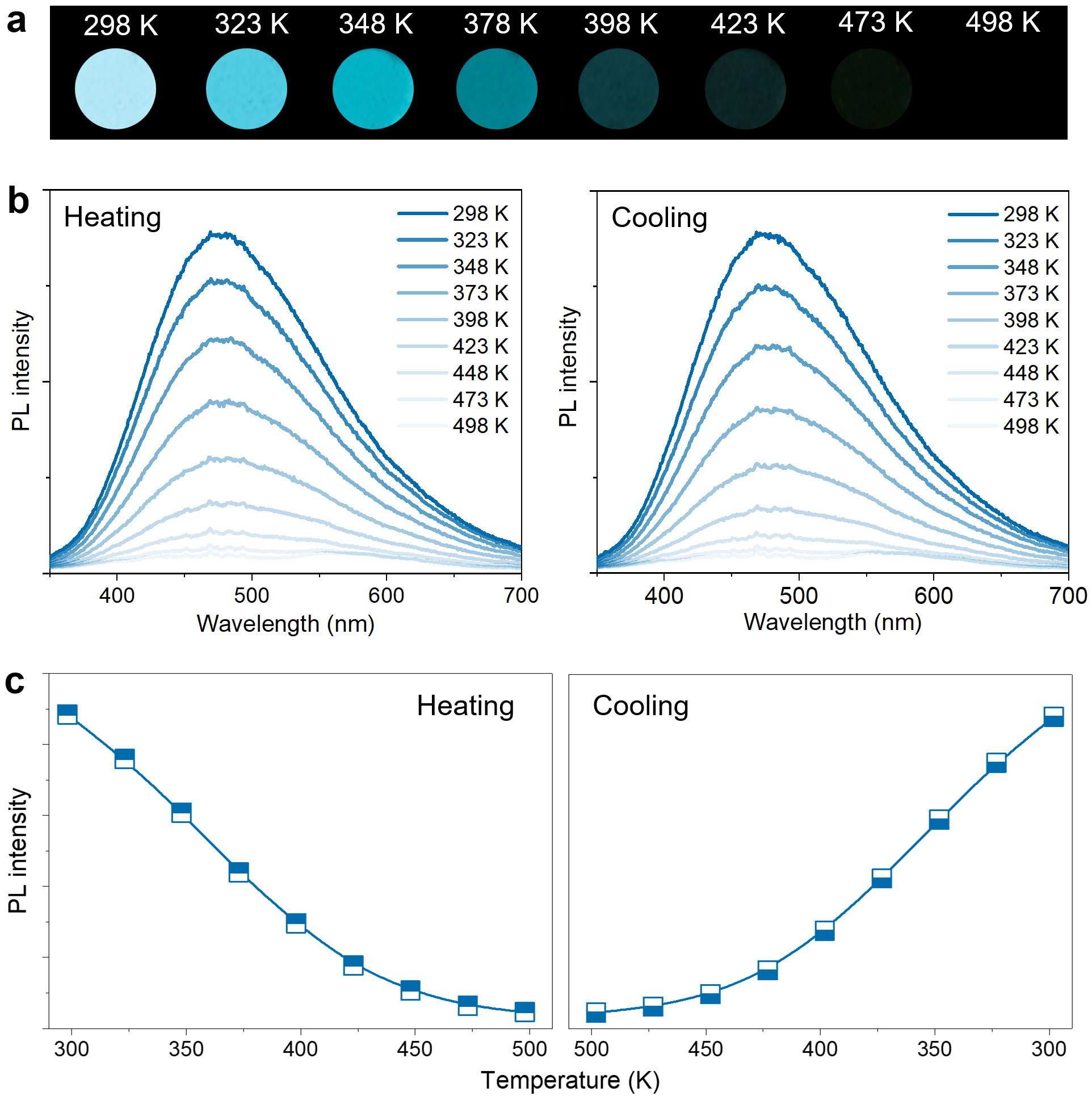


Supplementary Figure 15 | Temperature-dependent PL properties of CaGa_4_O_7_:Mn^2+^. a, Photographs of CaGa_4_O_7_ under photoexcitation when heated from 298 K to 498 K. b, PL spectra of CaGa_4_O_7_ when heated from 298 K to 498 K (left), and subsequently cooled to 298 K (right). c, Relative PL intensity of host emission when heated from 298 K to 498 K (left), and subsequently cooled to 298 K (right). The blue emission of the CaGa_4_O_7_ host shows a typical characteristic of thermal quenching. No detectable spectral shift was observed in the heating-cooling cycle. Furthermore, the decreased PL intensity during heating returns to its previous value as the temperature decreases. This thermal quenching phenomenon is ascribed to the vibration aggravation with temperature, which promotes the domination of nonradiative multiphonon transition probability.


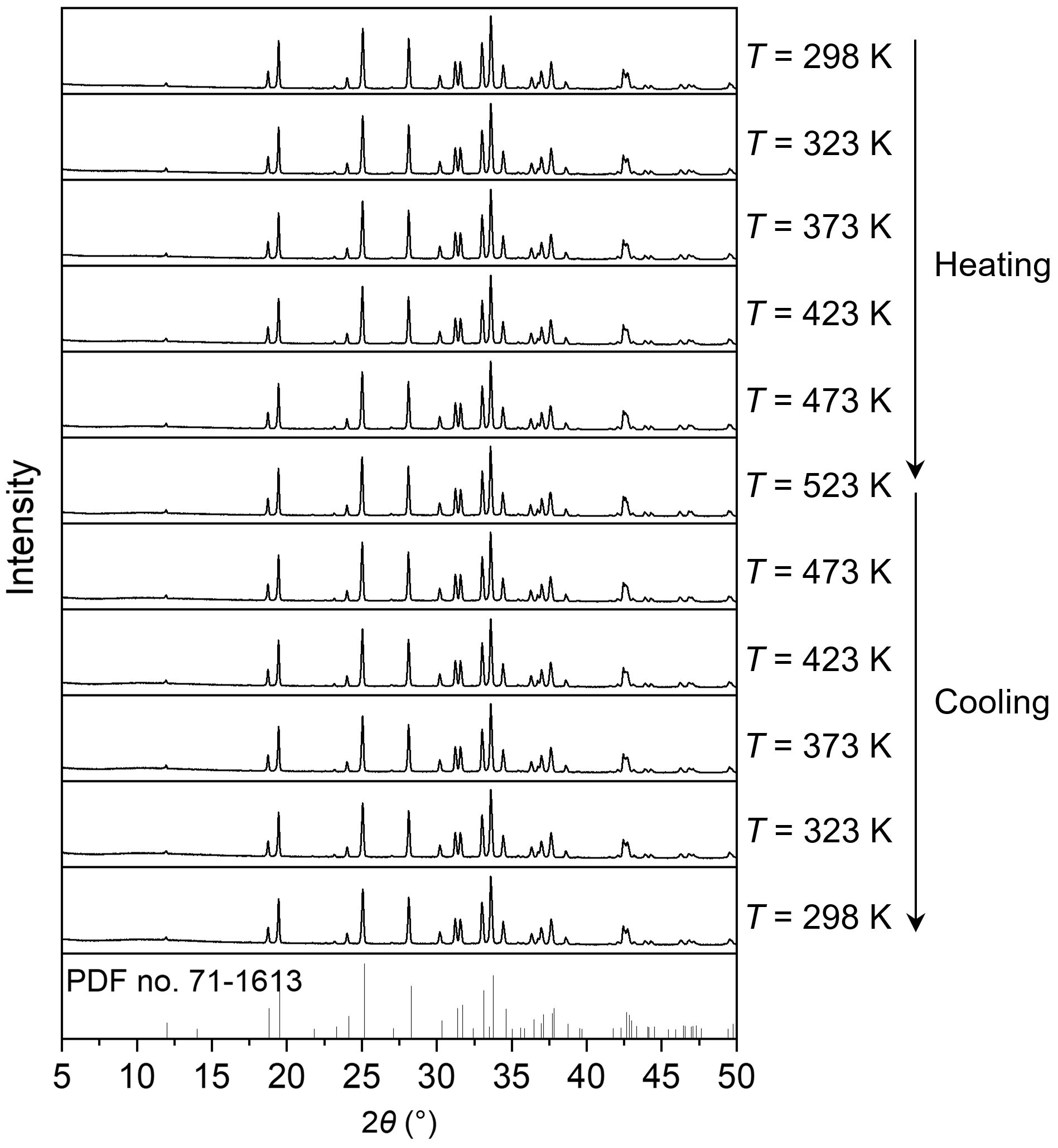


Supplementary Figure 16 | Temperature-dependent XRD patterns of CaGa_4_O_7_:Mn^2+^. The sample was heated from 298 K to 523 K and subsequently cooled to 298 K. The powder diffraction file (PDF) is no. 71-1613. The results show that no phase transition occurred in the tested temperature range.


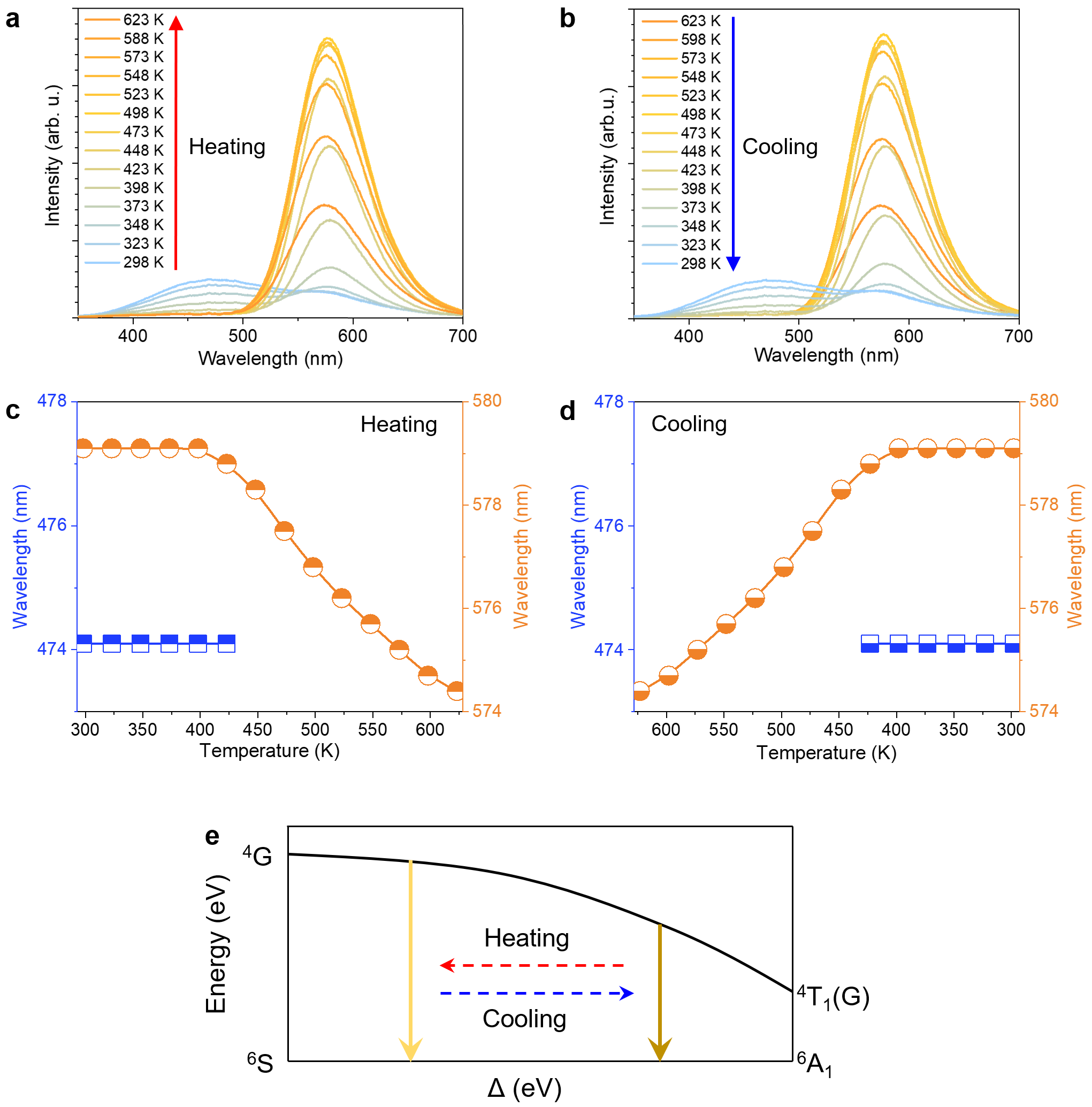


Supplementary Figure 17 | Temperature-dependent spectral shift of Mn^2+^ emission. a,b, PL spectra of CaGa_4_O_7_:Mn^2+^ when heated from 298 K to 623 K, and subsequently cooled to 298 K. c,d, Temperature dependence of the peak wavelengths of blue emission (blue squares) and yellow emission (yellow cycles) during heating and subsequent cooling. e, Phenomenological model of the Tanabe−Sugano diagram for the Mn^2+^ ion. These results show that the blue emission has a fixed peak center at 474 nm regardless of the temperature change in the range of 298−423 K. In contrast, the peak location of the yellow emission is blue-shifted when heated from 298 K to 623 K, while red-shifted to the initial location when cooled to 298 K, showing excellent reproducibility. The peak location of the yellow emission shifts by less than 5 nm, which is difficult to distinguish with the naked eye. The spectral shift of the yellow emission is ascribed to the change in the crystal-field strength. During heating, the lattice expands and the crystal-field strength around the Mn^2+^ ions reduces, resulting in a blue shift of the spectra. During cooling, the lattice shrinks, and the crystal-filed strength around the Mn^2+^ activators increases, leading to a red shift of the spectra.


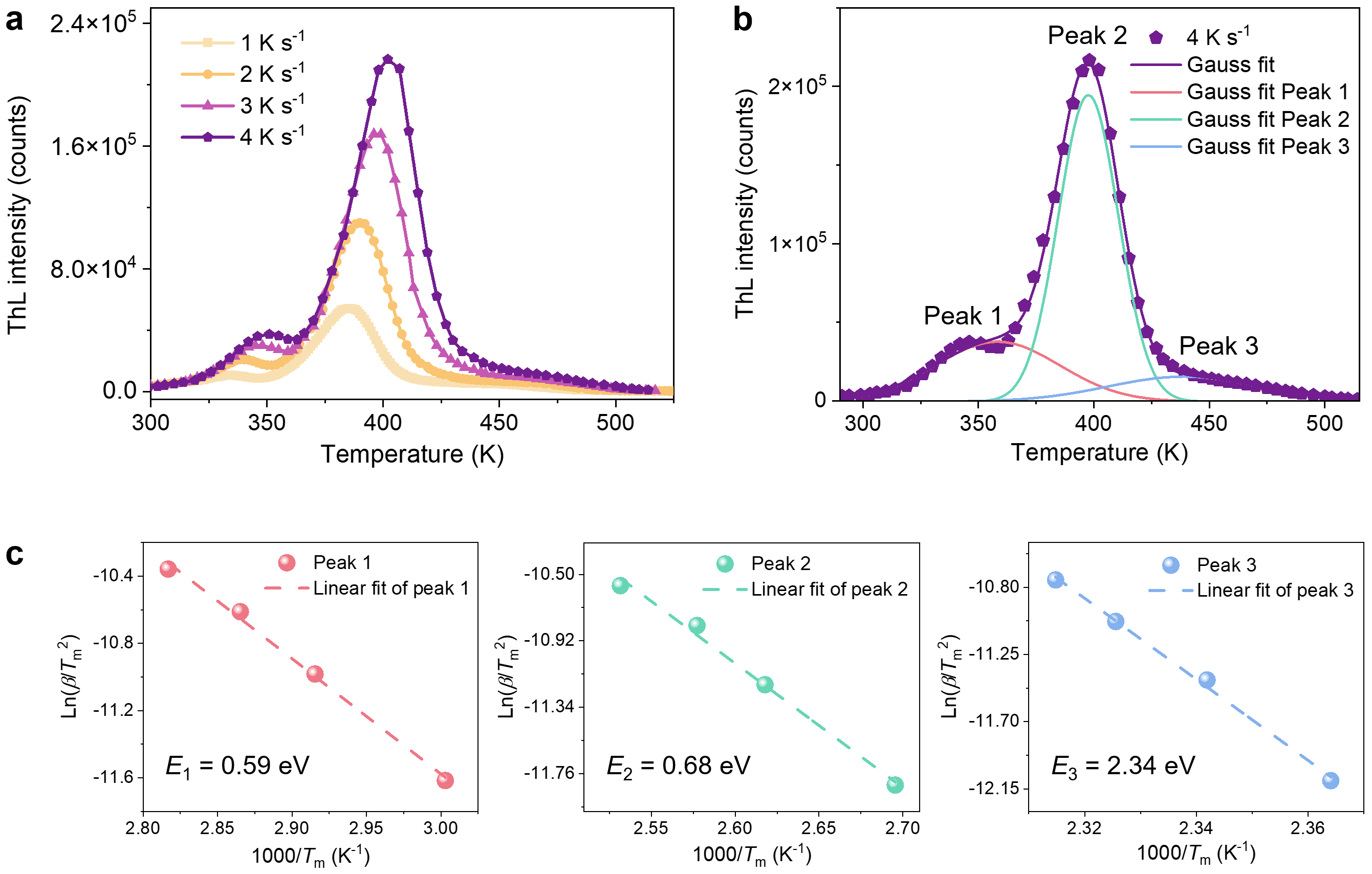


Supplementary Figure 18 | Calculation of trap depth based on ThL curves. a, ThL curves of CaGa_4_O_7_:Mn^2+^ obtained at different heating rates: 1, 2, 3, and 4 K s^−1^. b, Gaussian deconvolution of the ThL curve for CaGa_4_O_7_:Mn^2+^ obtained at a heating rate of 4 K s^−1^. c, Hoogenstraaten plots and derived trap depths for three ThL subpeaks. The trap depths were estimated to be 0.59, 0.68 and 2.34 for Peak 1, Peak 2 and Peak 3, respectively.

Supplementary Note 4. Studies of defect states


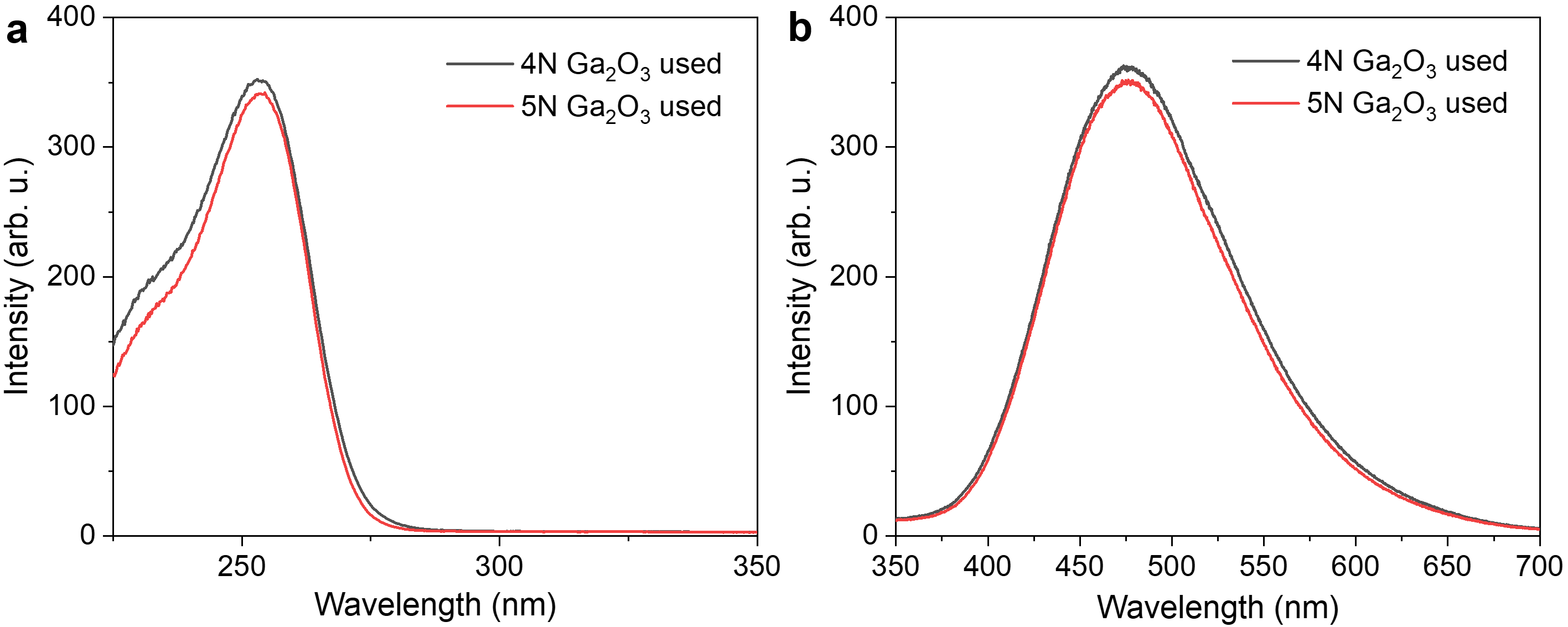


Supplementary Figure 19 | PLE and PL spectra of undoped CaGa_4_O_7_ prepared from Ga_2_O_3_ raw materials with different purities (99.99% and 99.999%). a, PLE spectra (*λ*_em_ = 474 nm). b, PL spectra (*λ*_ex_ = 254 nm). The results show that the intensity of blue emission diminished when a higher purity of Ga_2_O_3_ was used, suggesting that the unintentionally doped impurities in the material contribute to the blue emission of the host.


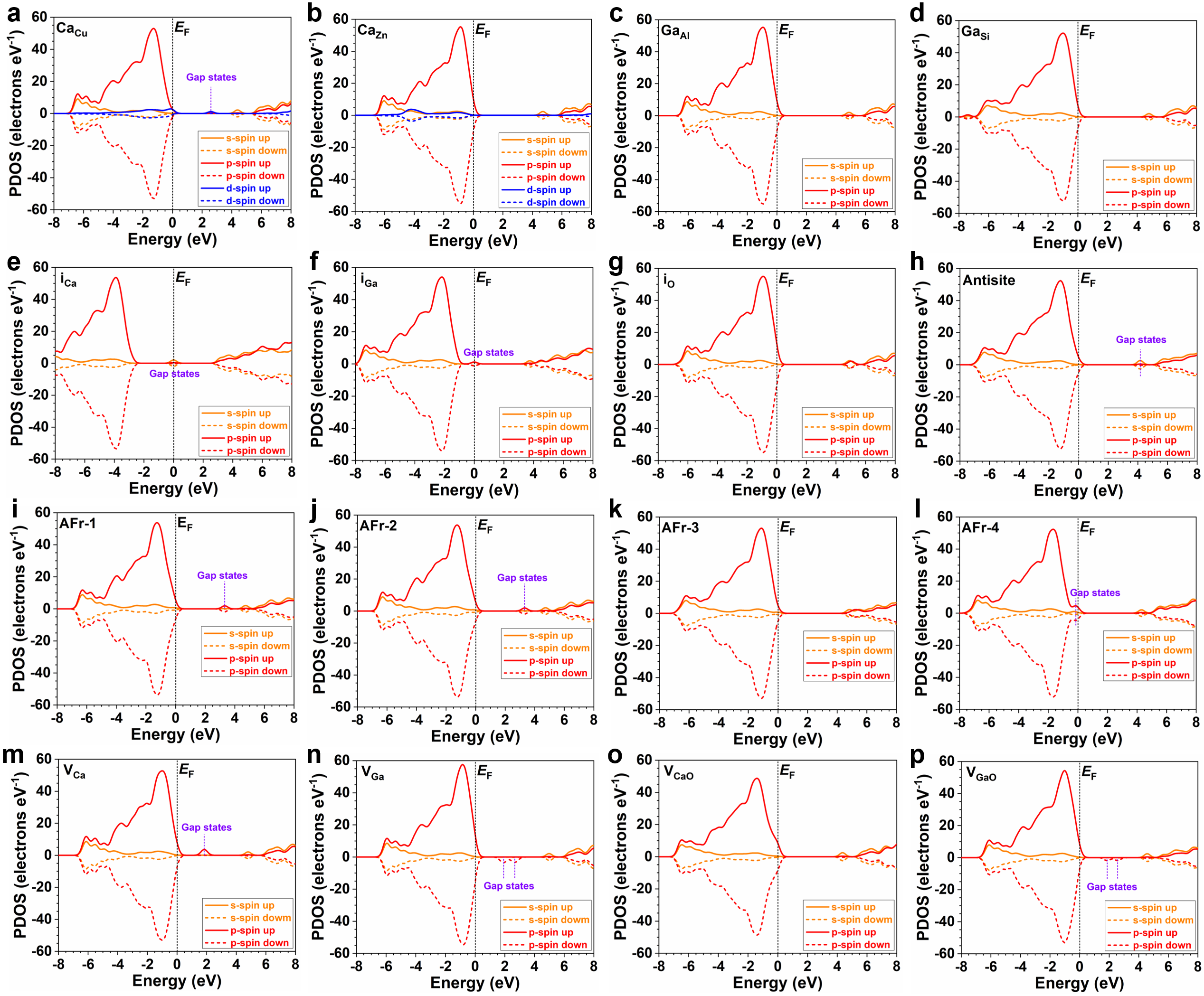


**Supplementary Figure 20 |** **The PDOSs of CaGa_4_O_7_ with different** **defects. a,** Cu impurity. **b,** Zn impurity. **c,** Al impurity. **d,** Si impurity. **e,** i_Ca_. **f,** i_Ga_. **g,** i_O_. **h,** Cation antisite. **i−l,** Anion Frenkel at different positions. **m,** V_Ca_. **n,** V_Ga_. **o,** V_CaO_. **p,** V_GaO_.


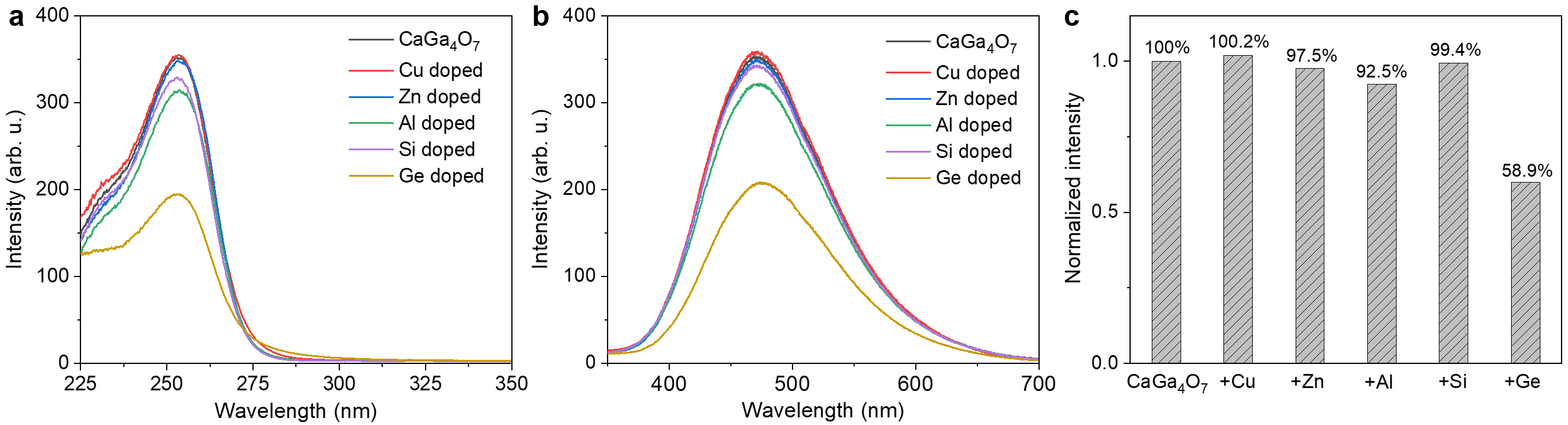


**Supplementary Figure 21 | PL properties of CaGa_4_O_7_ intentionally doped by Cu, Zn, Al, Si, and Ge (0.01 mol%) impurities. a,** Excitation spectra (*λ*_em_ = 474 nm). **b,** Emission spectra (*λ*_ex_ = 256 nm). **c,** Relative emission intensity of doped samples compared to undoped CaGa_4_O_7_. PL tests demonstrated that the introduction of impurities did not alter the profile and location of the excitation and emission peaks, but changed the intensity of these peaks in CaGa_4_O_7_. However, a noticeable change was observed in the intensity of these peaks. Specifically, Cu dopants resulted in a modest 0.2% increase in host emission, while Zn, Al, Si, and Ge dopants led to a reduction in emission intensity within the range of 0.6% to 40.1%. These experimental findings consistently align with theoretical calculations. These results underscore that even trace impurities have the capability to finely modulate the luminescence performance of CaGa_4_O_7_. Extensive sample preparation and systematic studies will be further carried out to optimize both the selection of elemental species and the appropriate doping amount.


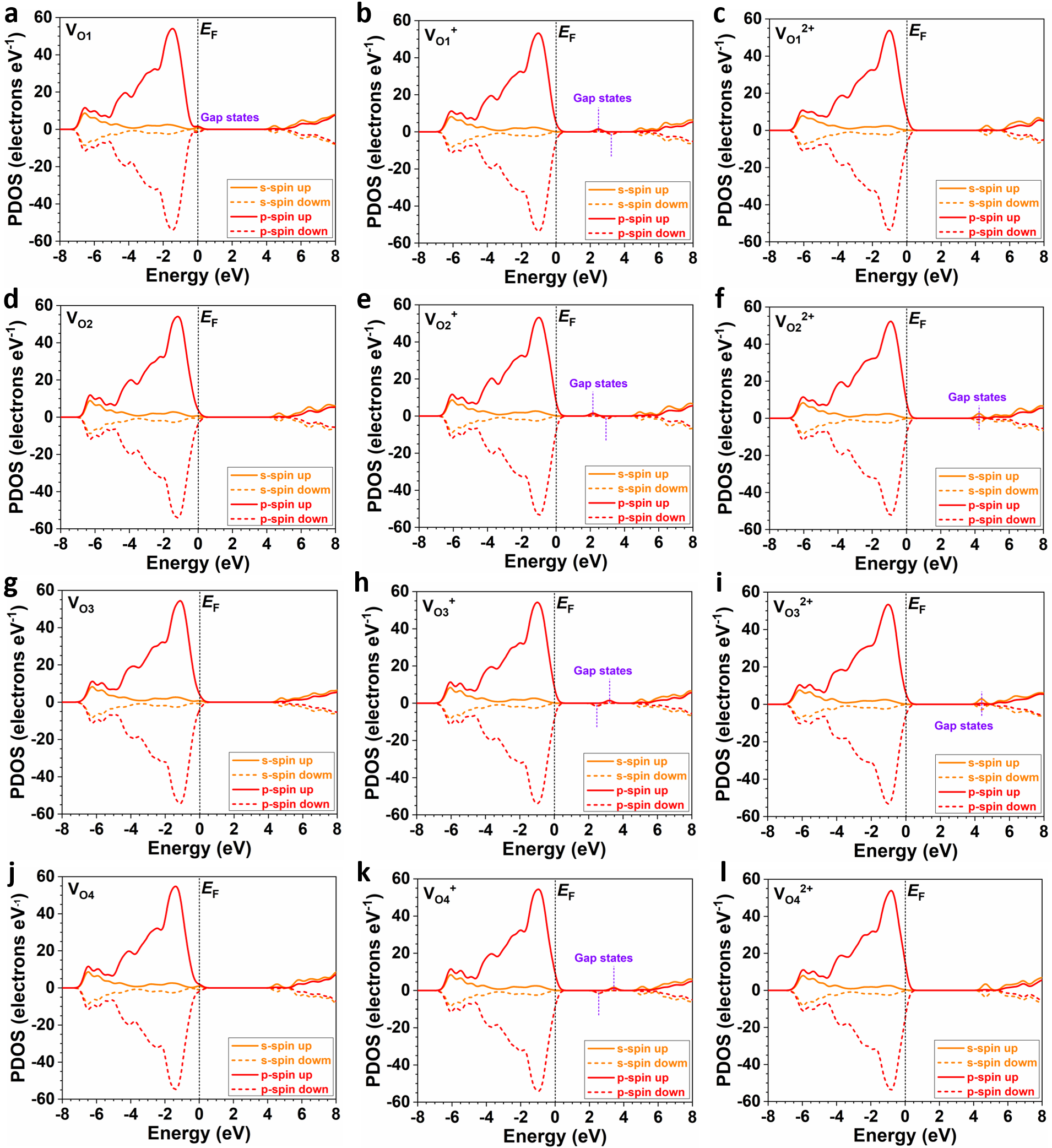


**Supplementary Figure 22 |** **The PDOSs of CaGa_4_O_7_ with oxygen vacancies. a,** V_O1_. **b,** V_O1_^+^. **c,** V_O1_^2+^. **d,** V_O2_. **e,** V_O2_^+^. **f,** V_O2_^2+^. **g,** V_O3_. **h,** V_O3_^+^. **i,** V_O3_^2+^. **j,** V_O4_. **k,** V_O4_^+^. **l,** V_O4_^2+^.


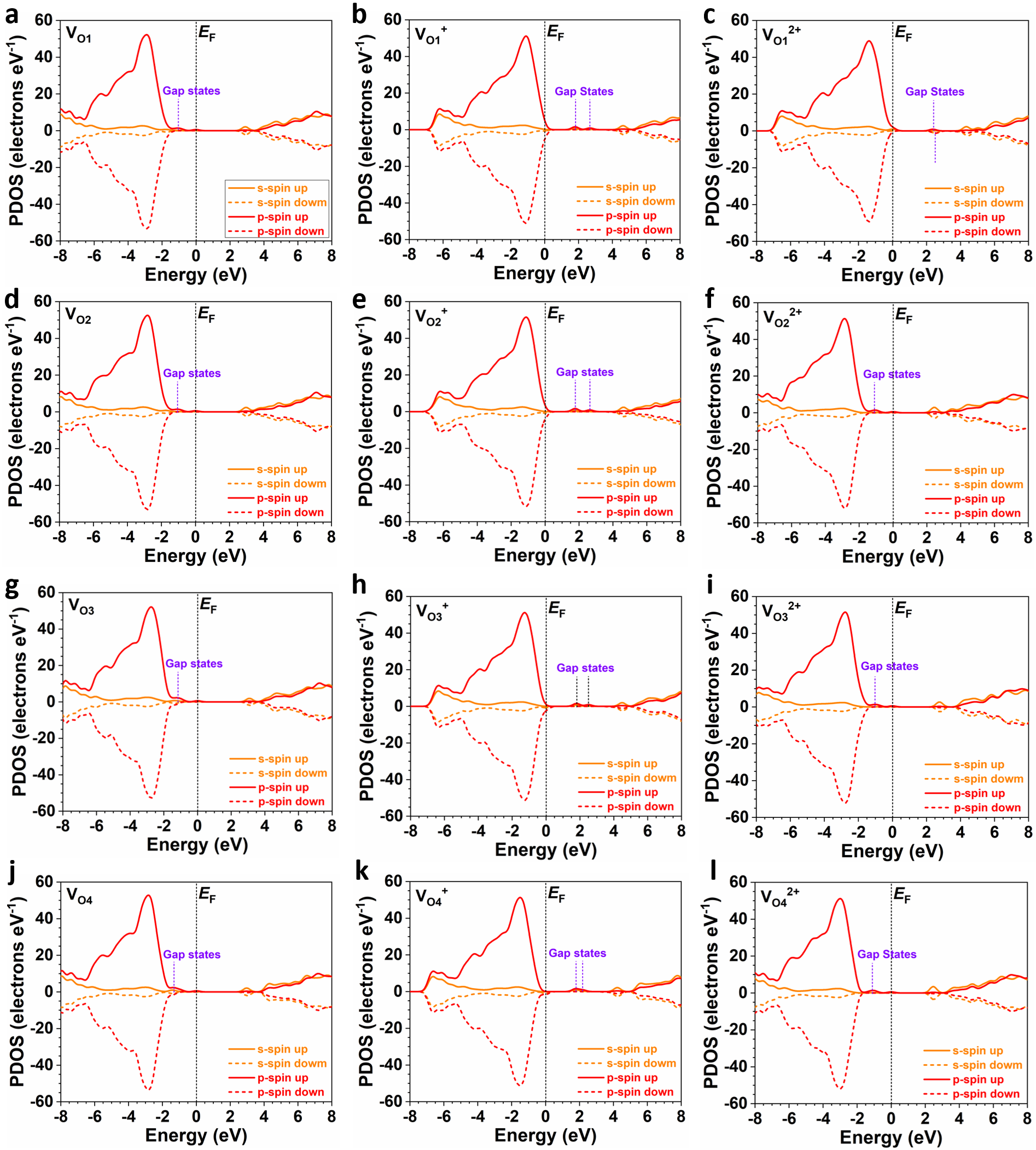


**Supplementary Figure 23 |** **The PDOSs of CaGa_4_O_7_:Mn^2+^ with oxygen vacancies. a,** V_O1_. **b,** V_O1_^+^. **c,** V_O1_^2+^. **d,** V_O2_. **e,** V_O2_^+^. **f,** V_O2_^2+^. **g,** V_O3_. **h,** V_O3_^+^. **i,** V_O3_^2+^. **j,** V_O4_. **k,** V_O4_^+^. **l,** V_O4_^2+^.


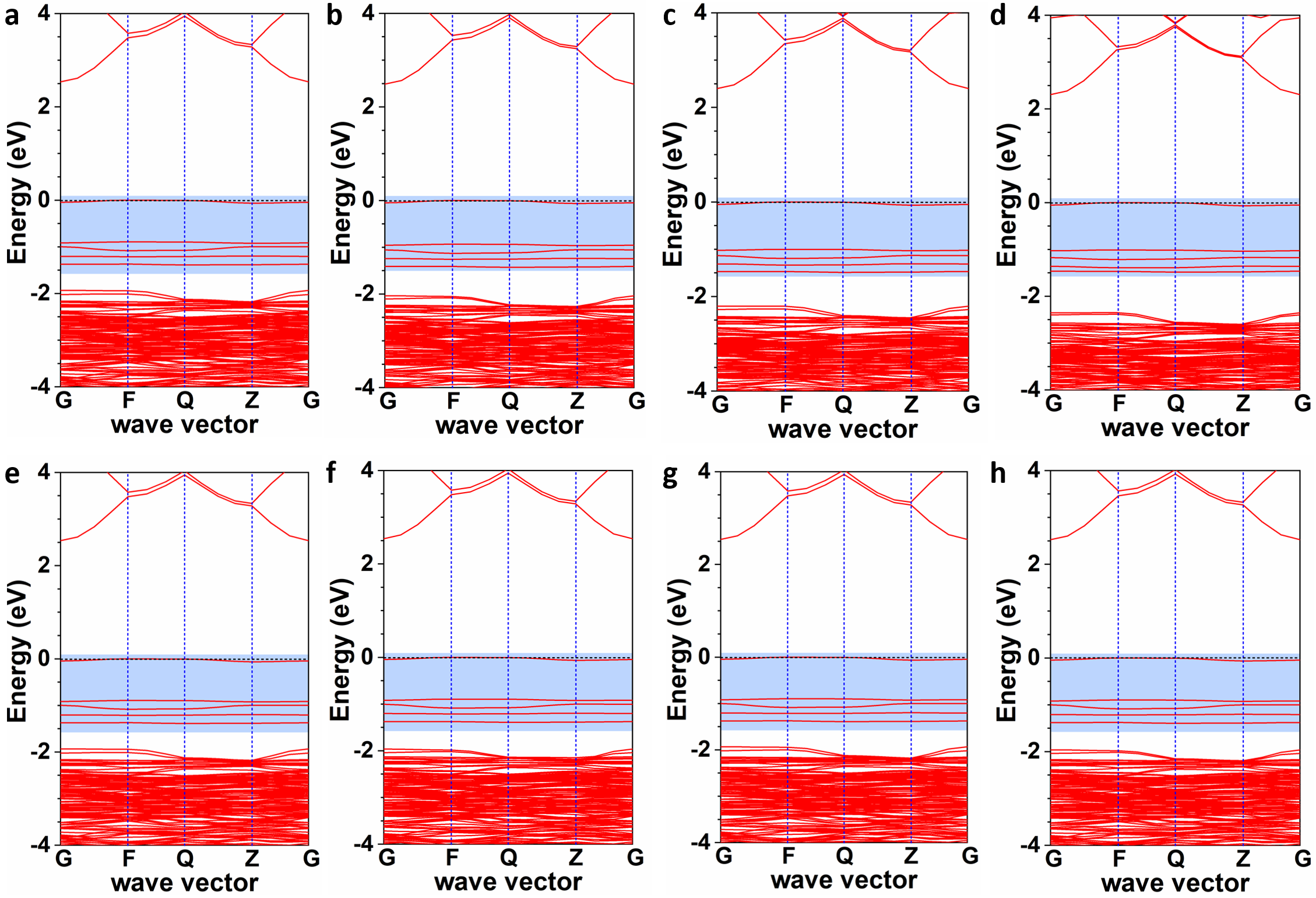


**Supplementary Figure 24 |** **The bandstructure of CaGa_4_O_7_:Mn^2+^ with external strain and stress. a−d,** Strain: (a) 0%, (b) 1%, (c) 3%, and (d) 5% on the (001) direction. **e−h,** Stress: (e) 0 GPa, (f) 10 GPa, (g) 30 GPa, and (h) 50 GPa.

Supplementary Note 5. Physics and interactions in dynamic multimodal luminescence


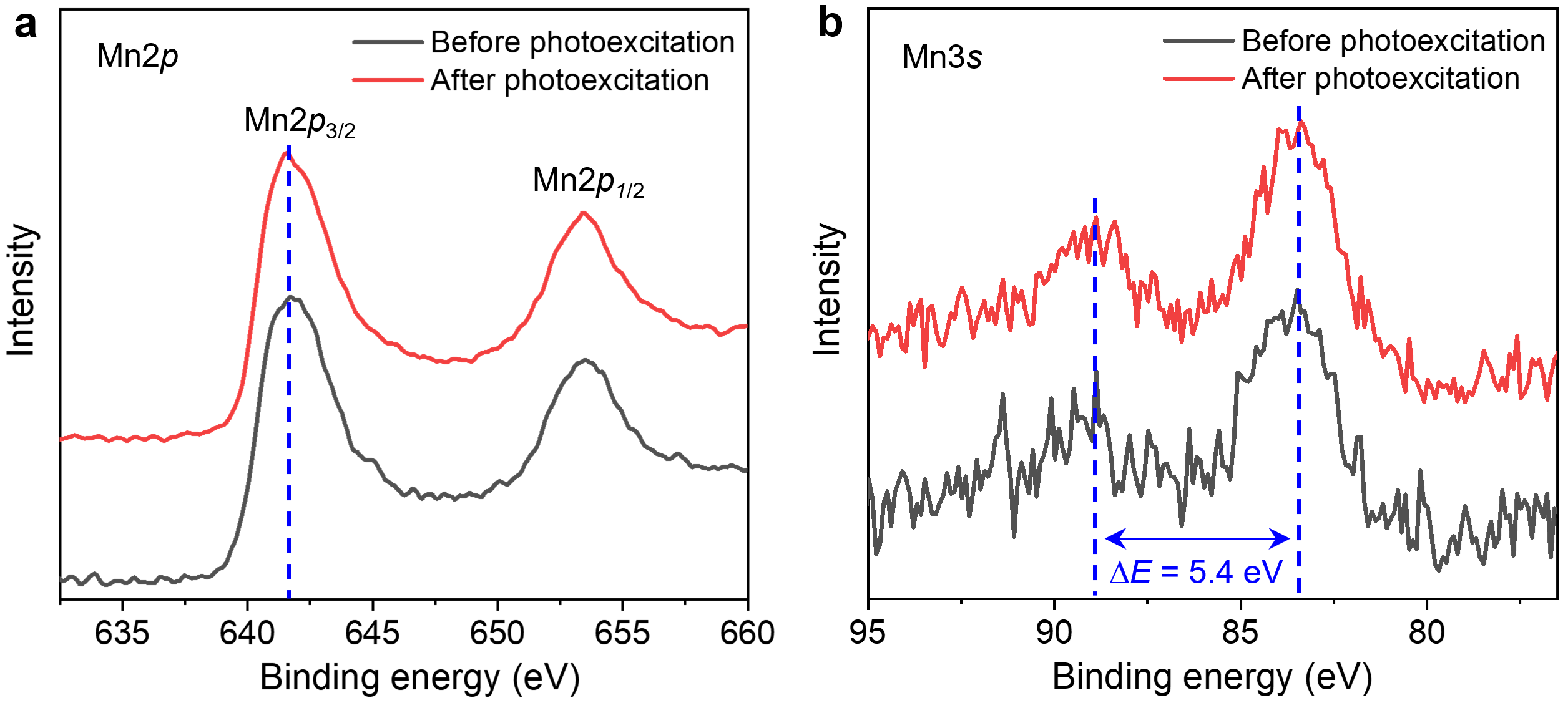


Supplementary Figure 25 | XPS spectra of Ca_1−_*_x_*Ga_4_O_7_:*x*Mn^2+^ (*x* = 1×10^−4^) before and after photoexcitation. a, Mn2*p* spectra. b, Mn3*s* spectra.


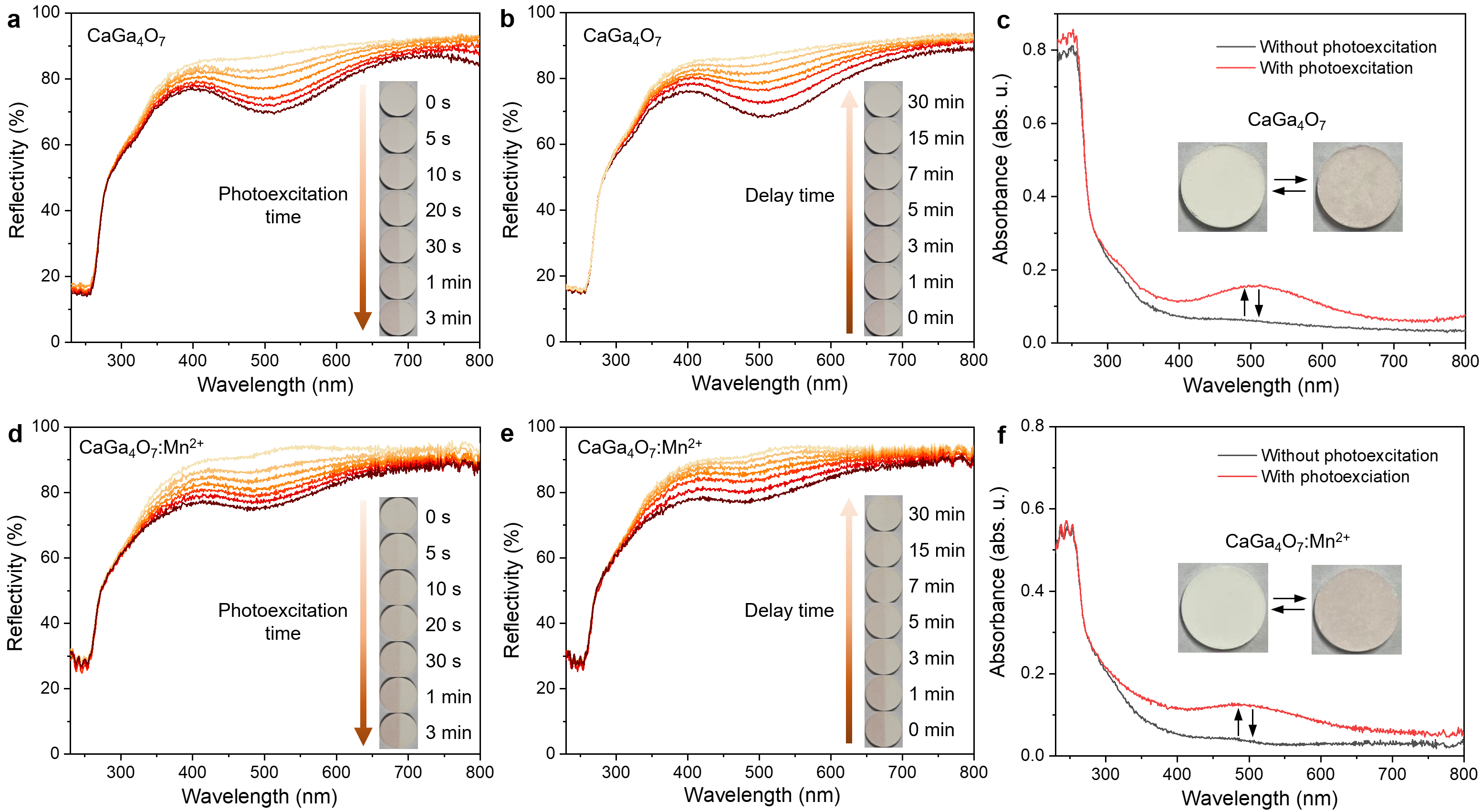


**Supplementary Figure 26 | Photochromic coloring and decolorization processes of CaGa_4_O_7_ and CaGa_4_O_7_:Mn^2+^. a,d,** Diffuse reflectance spectra and photochromic photographs of CaGa_4_O_7_ and CaGa_4_O_7_:Mn^2+^ disks under 254 nm irradiation for different photoexcitation time (0 s−3 min). **b,e,** Diffuse reflectance spectra and photochromic photographs of CaGa_4_O_7_ and CaGa_4_O_7_:Mn^2+^ disks after 254 nm photoexcited (3 min) with different delay times (0−30 min). **c,f,** Absorption spectra and photochromic photographs of CaGa_4_O_7_ and CaGa_4_O_7_:Mn^2+^ disks without/with photoexcitation (254 nm, 3 min). The results show that the CaGa_4_O_7_ and CaGa_4_O_7_:Mn^2+^ disks exhibit similar photochromic phenomena under UV irradiation, and the apparent color of both materials changes significantly from milky white to brown. Moreover, the absorption peaks of the two materials after photoexcitation show the same peak position, which indicates that the photochromic phenomena of both originate from the same color centers.


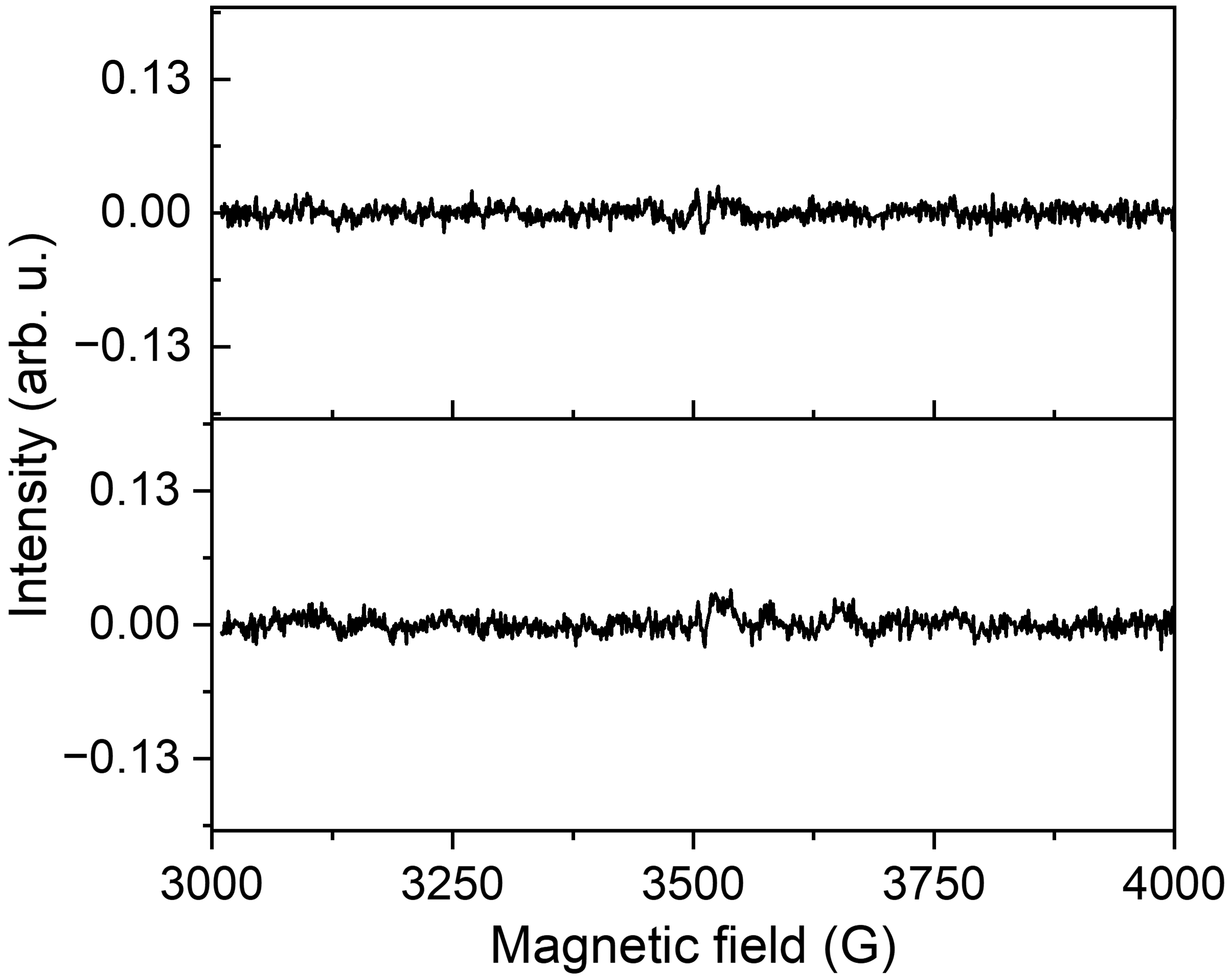


**Supplementary Figure 27 | EPR spectra of** **Ca_1_**_−_***_x_*Ga_4_O_7_:*x*Mn^2+^ (*x* = 1×10**^−^**^4^ and 10×10**^−^**^4^).** The results show that no characteristic signals of Mn^2+^ ions were detected due to low concentration doping of Mn^2+^ ions and no signals of Mn^4+^ were found.


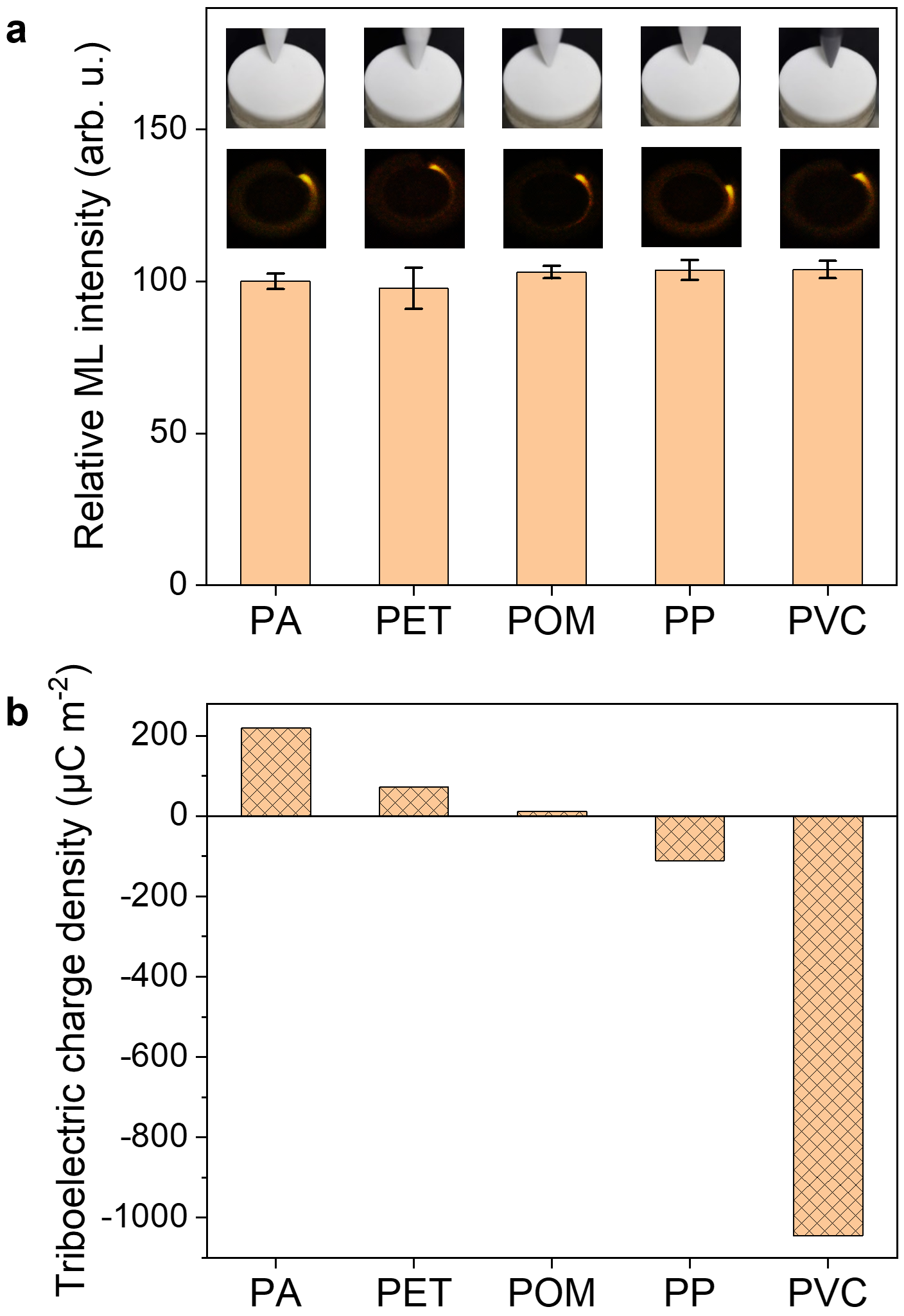


Supplementary Figure 28 | ML properties of CaGa_4_O_7_:Mn^2+^ composites under mechanical friction. a, Comparison of the ML intensity of CaGa_4_O_7_:Mn^2+^ composites excited by friction rods of five materials. PA = polyamide, PP = polypropylene, POM = polyformaldehyde, PET = polyethylene terephthalate, PVC = polyvinyl chloride. Error bars represent standard deviation, n = 5 independent replicates. The insets show the photographs of the ML setup in a lighting room (upper) and ML images in a dark room (bottom). The CaGa_4_O_7_:Mn^2+^ composites were photoexcited by a 254 nm lamp for 1 min and then delayed for 10 s prior to the ML testing. The pressure of the friction is 4.81 MPa. b, Triboelectric charge density of the five materials. The values of triboelectric charge density are from the reference.^18^


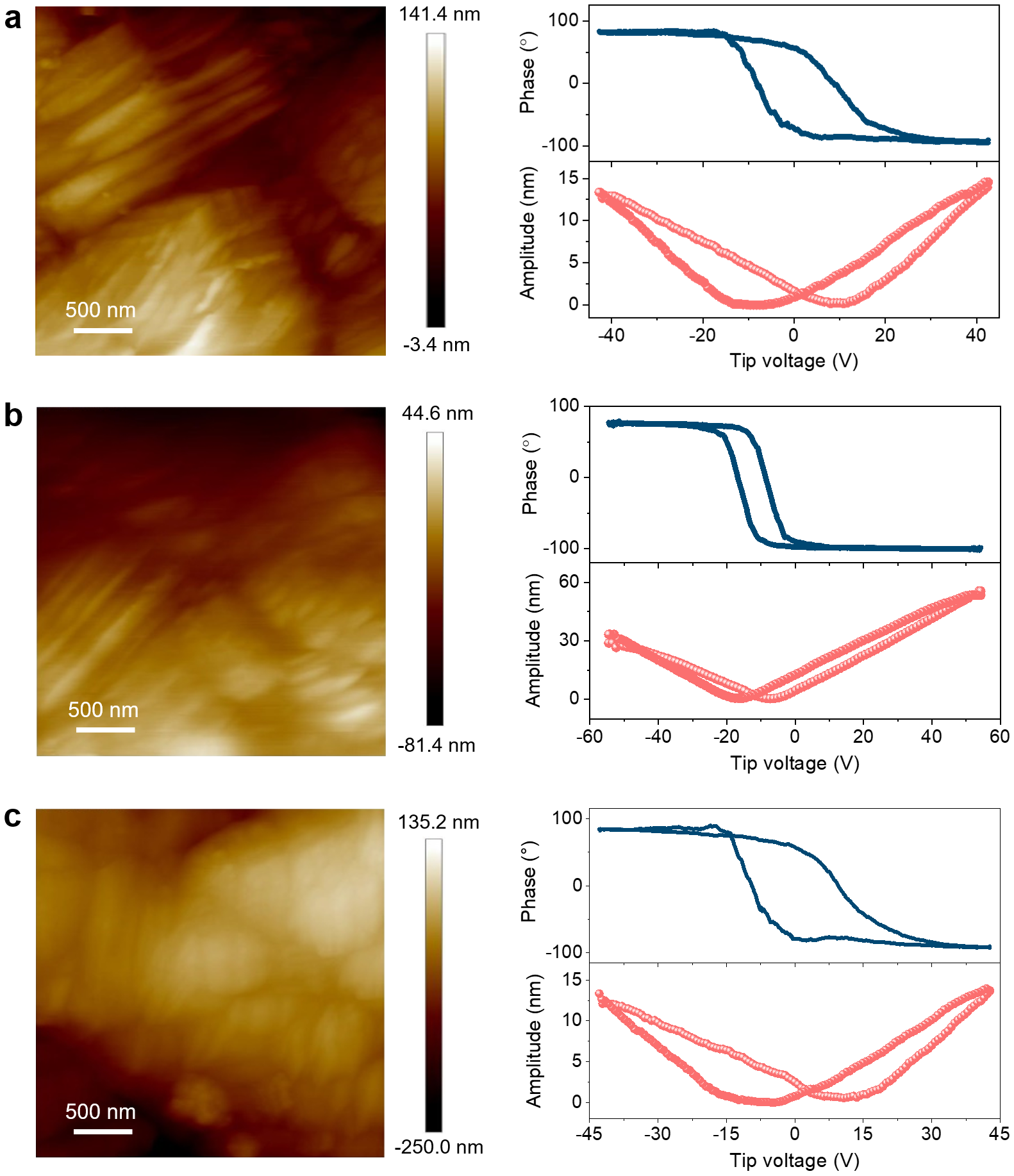


Supplementary Figure 29 | Piezoresponse force microscope measurements. a−c, PFM morphology images (left) and PFM hysteresis loops (right) of three randomly selected CaGa_4_O_7_:Mn^2+^ particles.

Supplementary Note 6. Applications in dynamic color and pattern displays under photo-thermo-mechanical stimulations


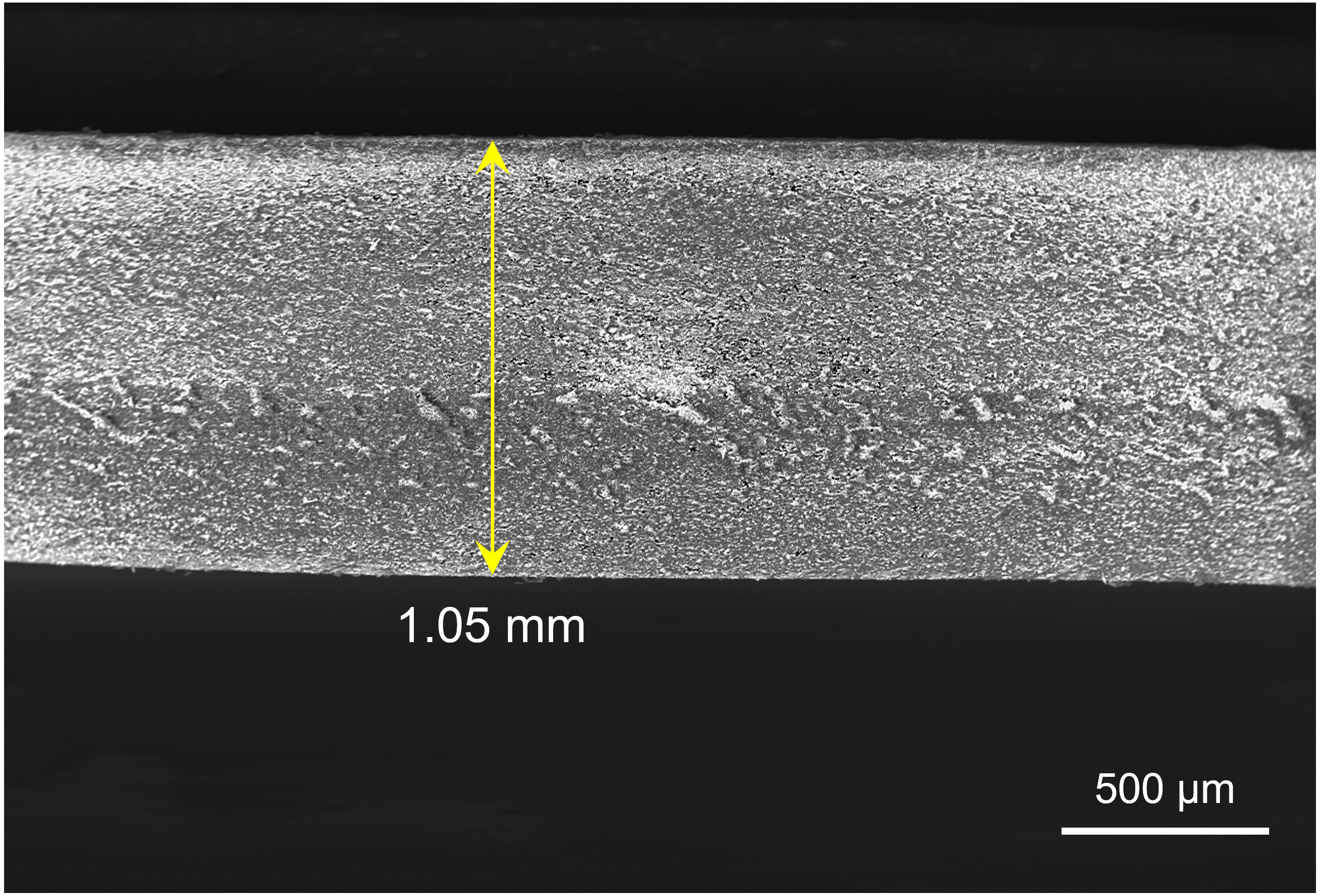


Supplementary Figure 30 | SEM image of the cross-section of the prepared composite film. It shows a thickness of approximately 1 mm.


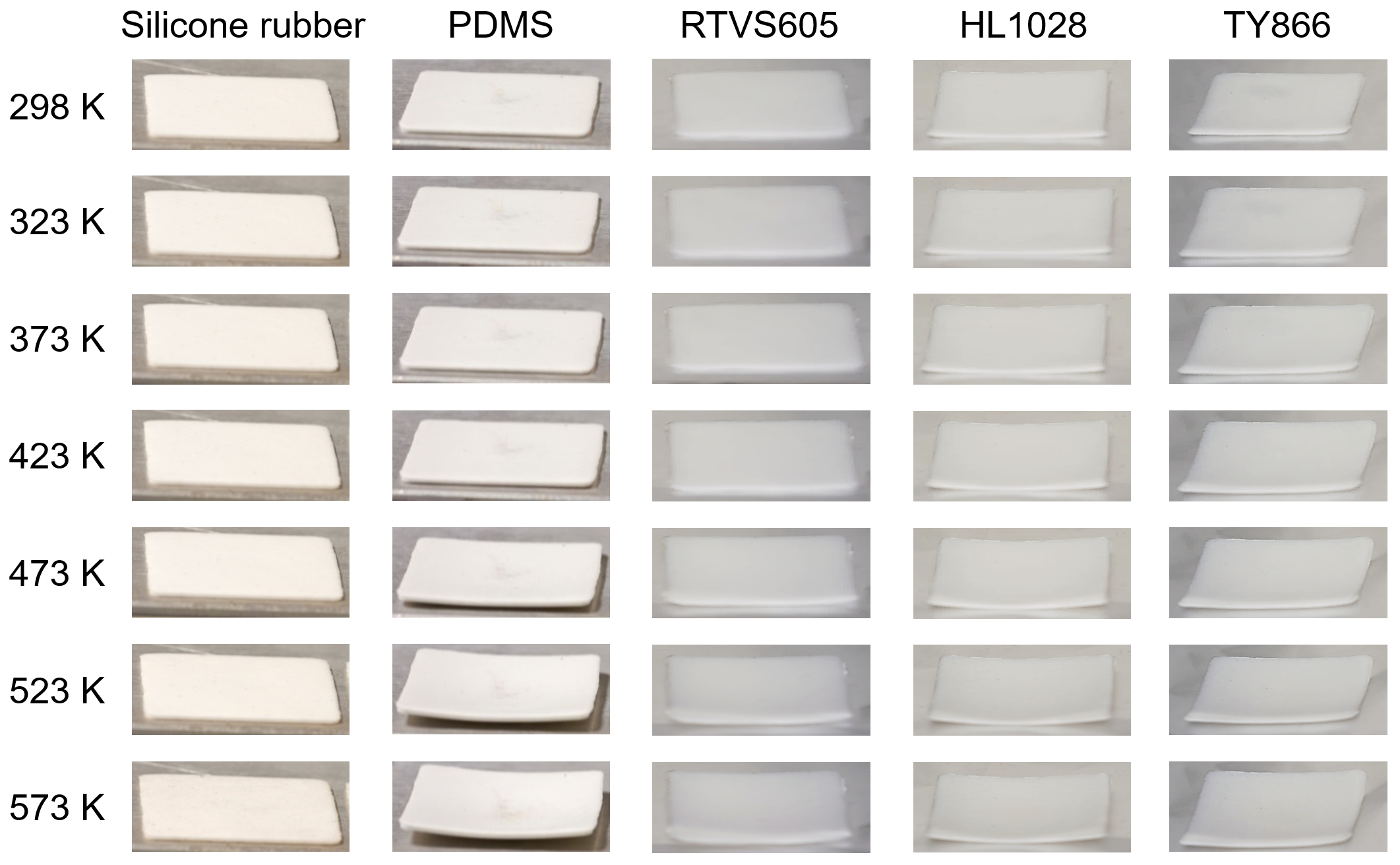


**Supplementary Figure 31 | Photographs of five CaGa_4_O_7_:Mn^2+^/polymer composite films at different temperatures (298–573 K).** The silicone rubber-based film remained flat with increasing temperature, while the other composite films curled above 473 K. These results indicate that the silicone rubber-based composite film has better thermal stability than the other composite films. The size of composite films is 40 × 40 × 1 mm^3^.


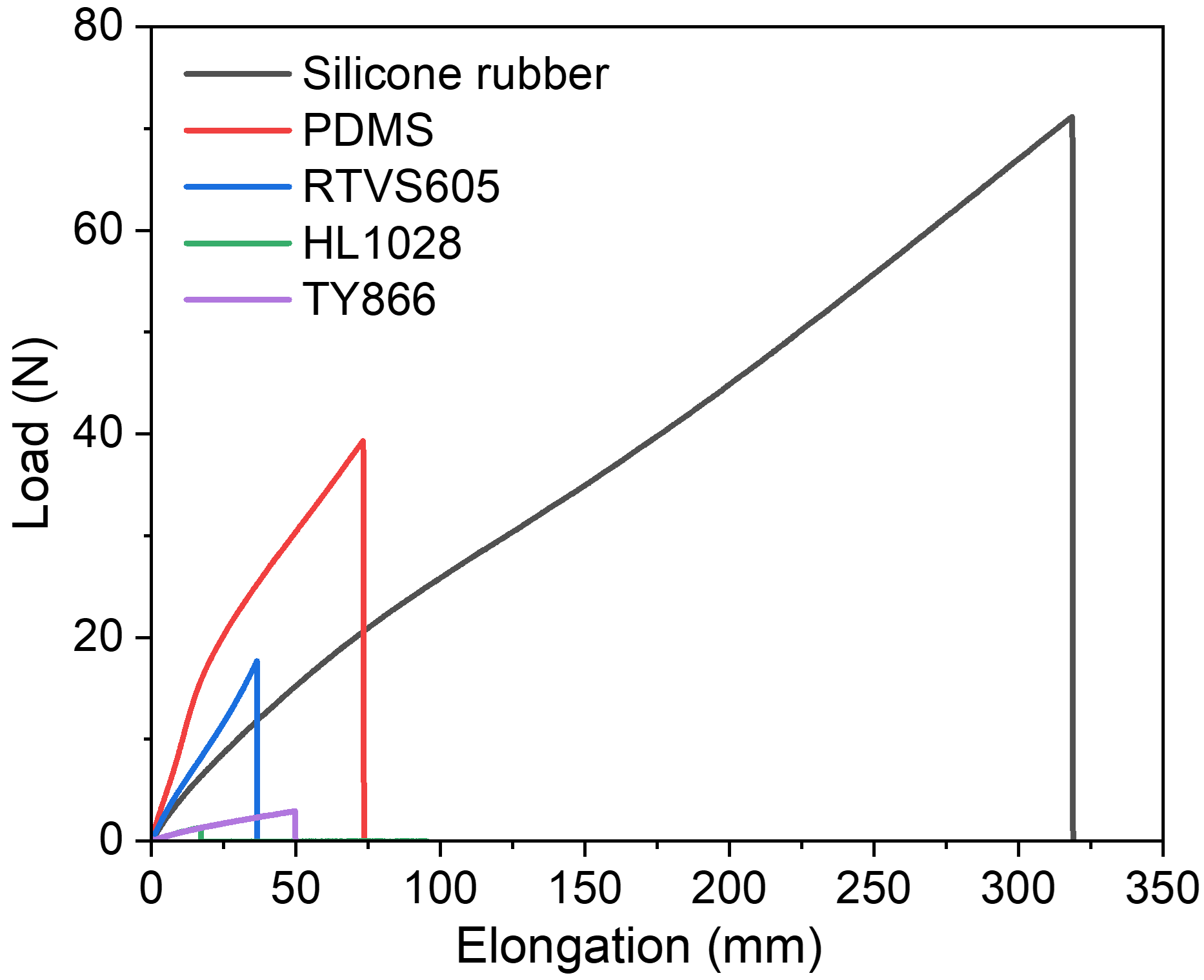


**Supplementary Figure 32 | Load-elongation curves of different CaGa_4_O_7_:Mn^2+^/polymer composite films.** It indicates higher stretchability for the silicone rubber-based composite.


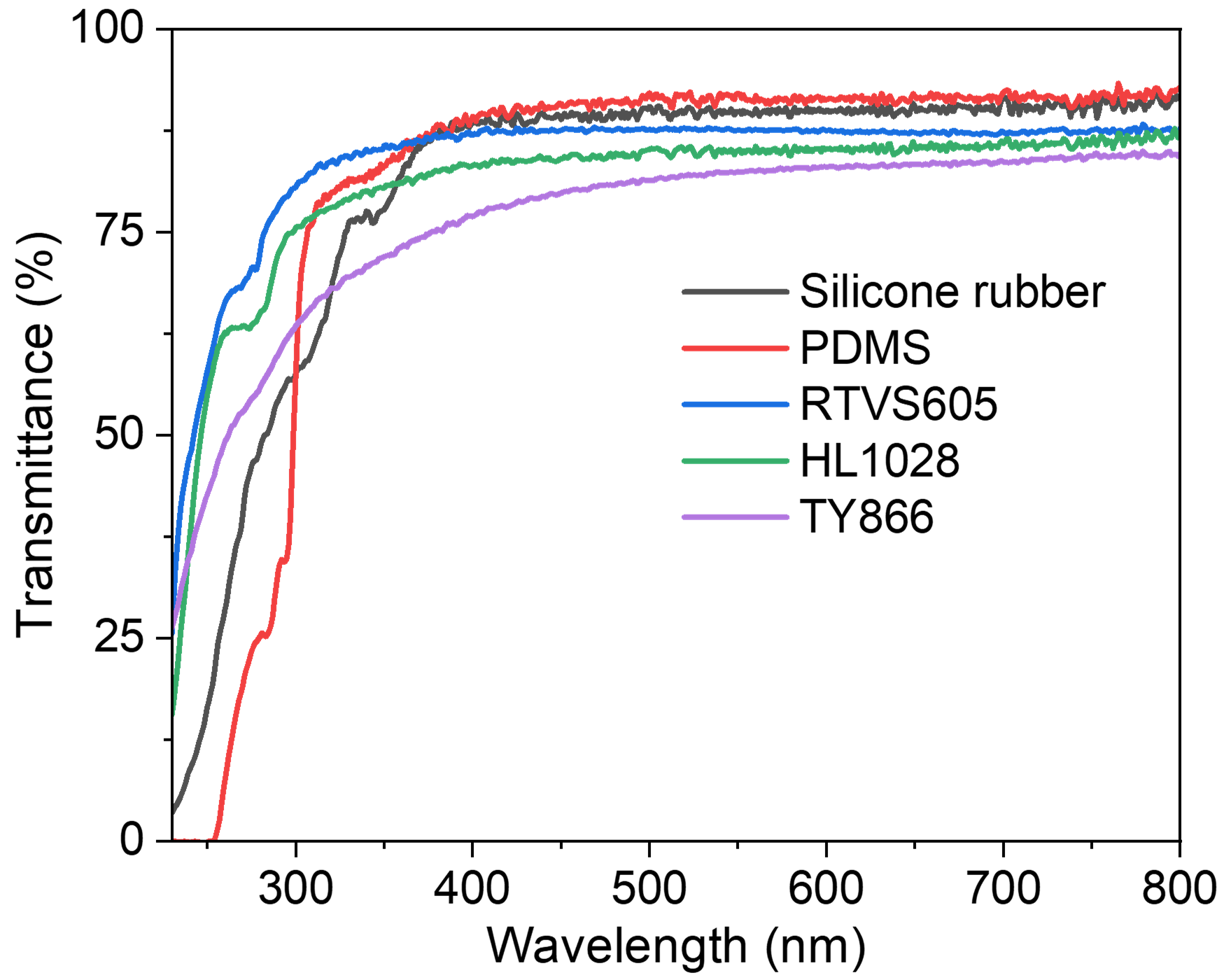


**Supplementary Figure 33 | Optical transmittance spectra of organic films synthesized from different materials.**


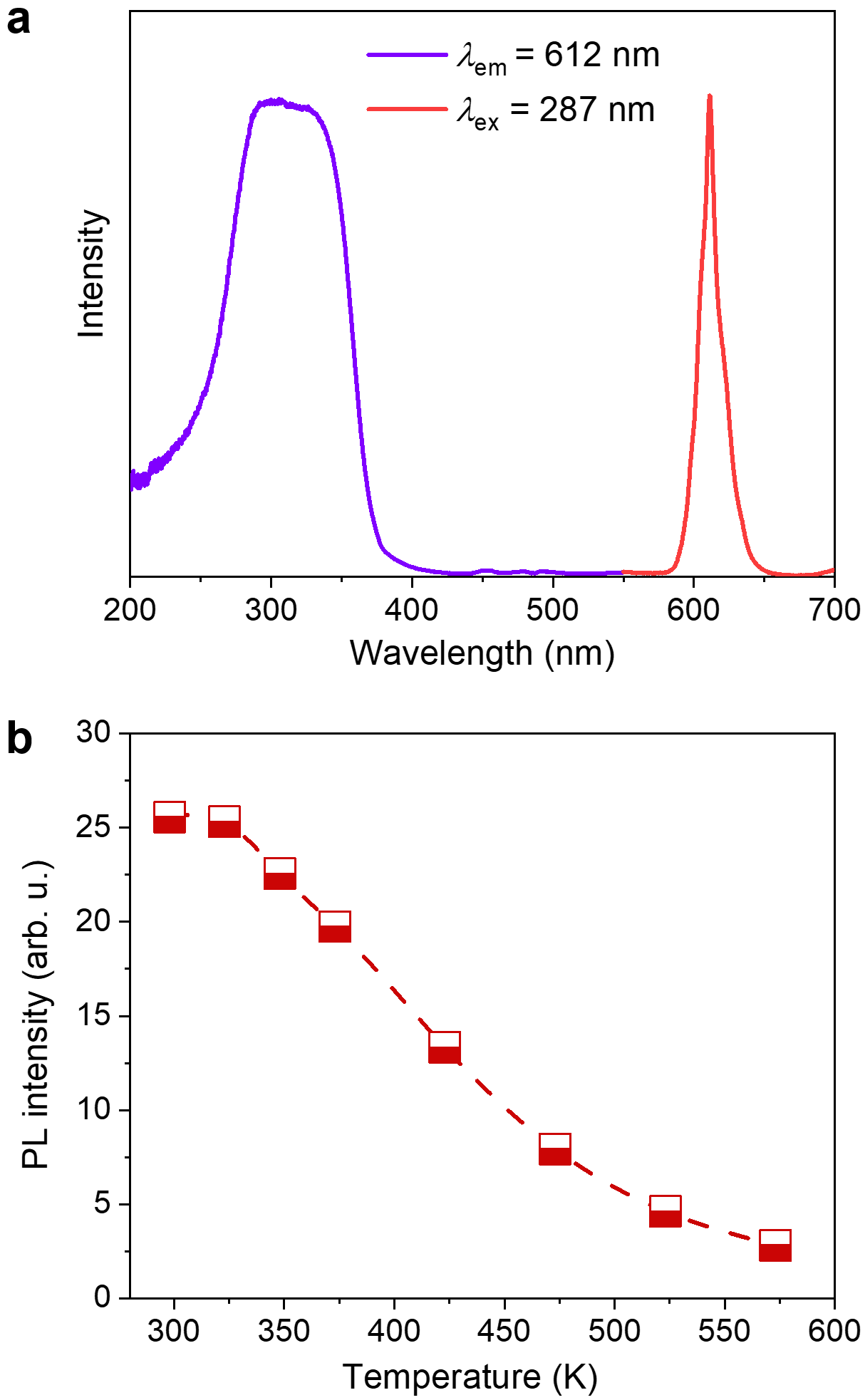


Supplementary Figure 34 | PL properties of NaNbO_3_:Pr^3+^. a, PL excitation and emission spectra of NaNbO_3_:Pr^3+^. The red emission centered at 612 nm originates from the optical transition of Pr^3+^ from ^1^D_2_ to ^3^H_4_. b, Relative PL intensity of NaNbO_3_:Pr^3+^ when heated from 298 to 573 K. It shows the property of thermal quenching.


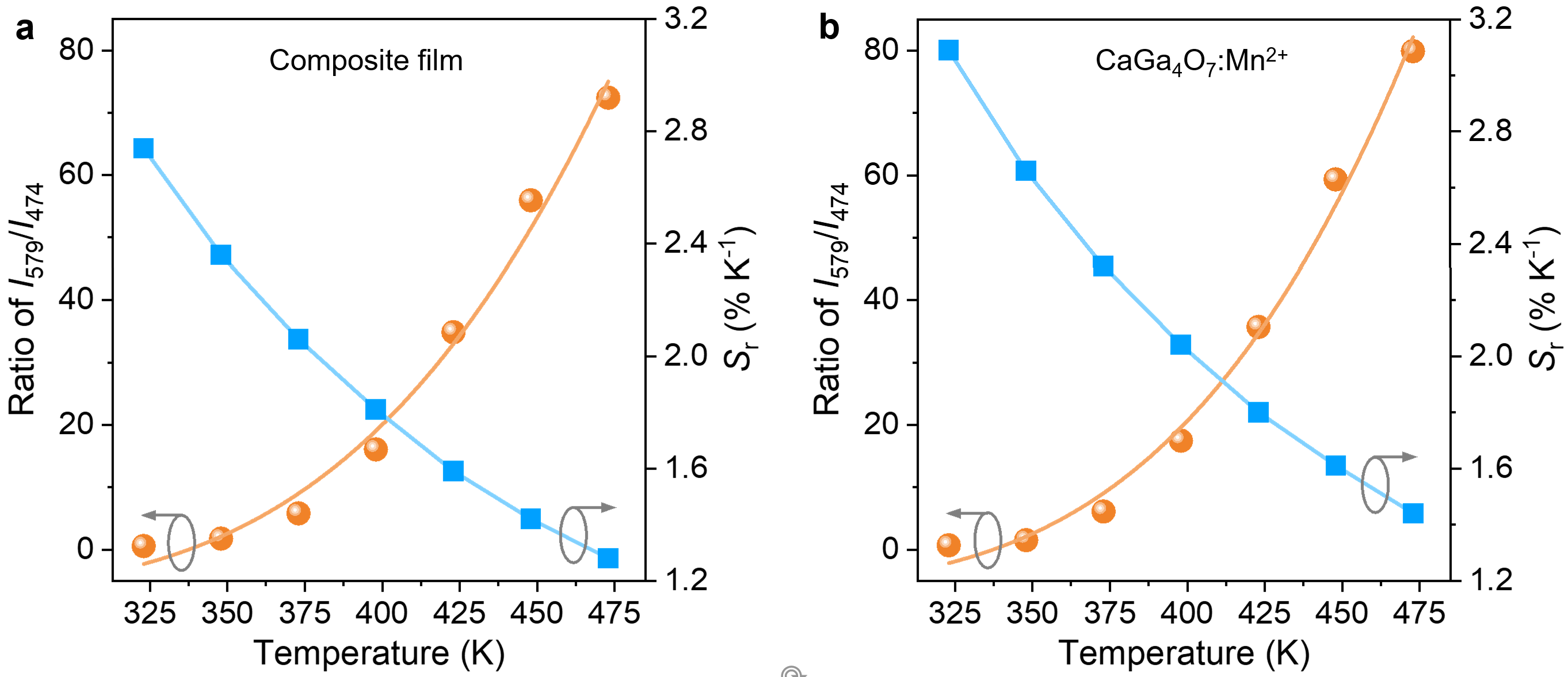


Supplementary Figure 35 | Temperature-dependent luminescent intensity ratio (*R*) and relative sensitivity (*S*_r_). a, CaGa_4_O_7_:Mn^2+^/PDMS composite film. *S_r_* =2.74% K^−1^ @323 K; 1.28% K^−1^ @473 K. b, CaGa_4_O_7_:Mn^2+^ phosphor devoid of polymer matrix. *S_r_* = 3.09% K^−1^ @323 K; 1.44% K^−1^ @473 K. The *S*_r_ values of the composite film are lower than those of the phosphor, which is due to the lower thermal conductivity of the polymer matrix.

The emission intensity ratios of *I*_579_/*I*_474_ versus the temperature for a CaGa_4_O_7_:Mn^2+^/PDMS composite film are well fitted as follows:

*R*(*I*_579_/*I*_474_) = 34839.57exp(−2860.73/*T*) − 7.27 (1)

The emission intensity ratios of *I*_579_/*I*_474_ versus the temperature for the CaGa_4_O_7_:Mn^2+^ phosphor devoid of polymer matrix are well fitted as follows:

*R*(*I*_579_/*I*_474_) = 57969.04exp(−3066.26/*T*) − 6.55 (2)

The relative sensitivity (*S_r_*) was calculated by the following equation^19^:

*S_r_* = $\frac{\boldsymbol{1}}{\boldsymbol{R}}\frac{\boldsymbol{dR}}{\boldsymbol{dT}}$ (3)


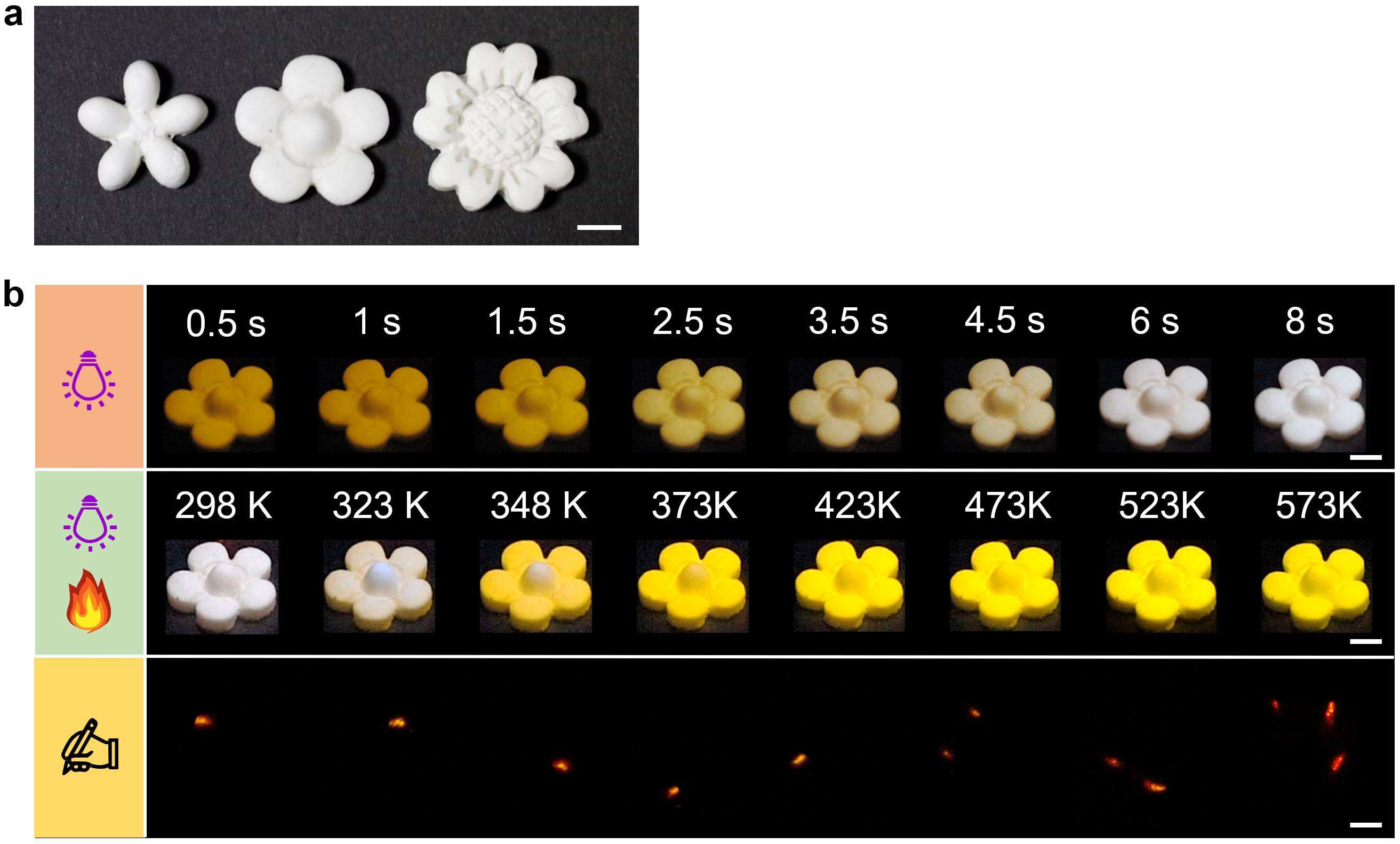


Supplementary Figure 36 | Multi-responsive multicolor luminescence of flower-shaped CaGa_4_O_7_:Mn^2+^ composites for multidimensional dynamic displays. a, Photographs of CaGa_4_O_7_:Mn^2+^/silicone rubber composite elastomers prepared into different three-dimensional shapes. b, Photographs of the luminescent responses of the flower-shaped elastomer under optical, thermal and mechanical stimulation. In the ML demonstration, ML was excited by pressing different petals of the flower-like elastomer, including pressing one of five petals separately, pressing two petals simultaneously, and pressing three petals simultaneously. Scale bar = 10 mm.


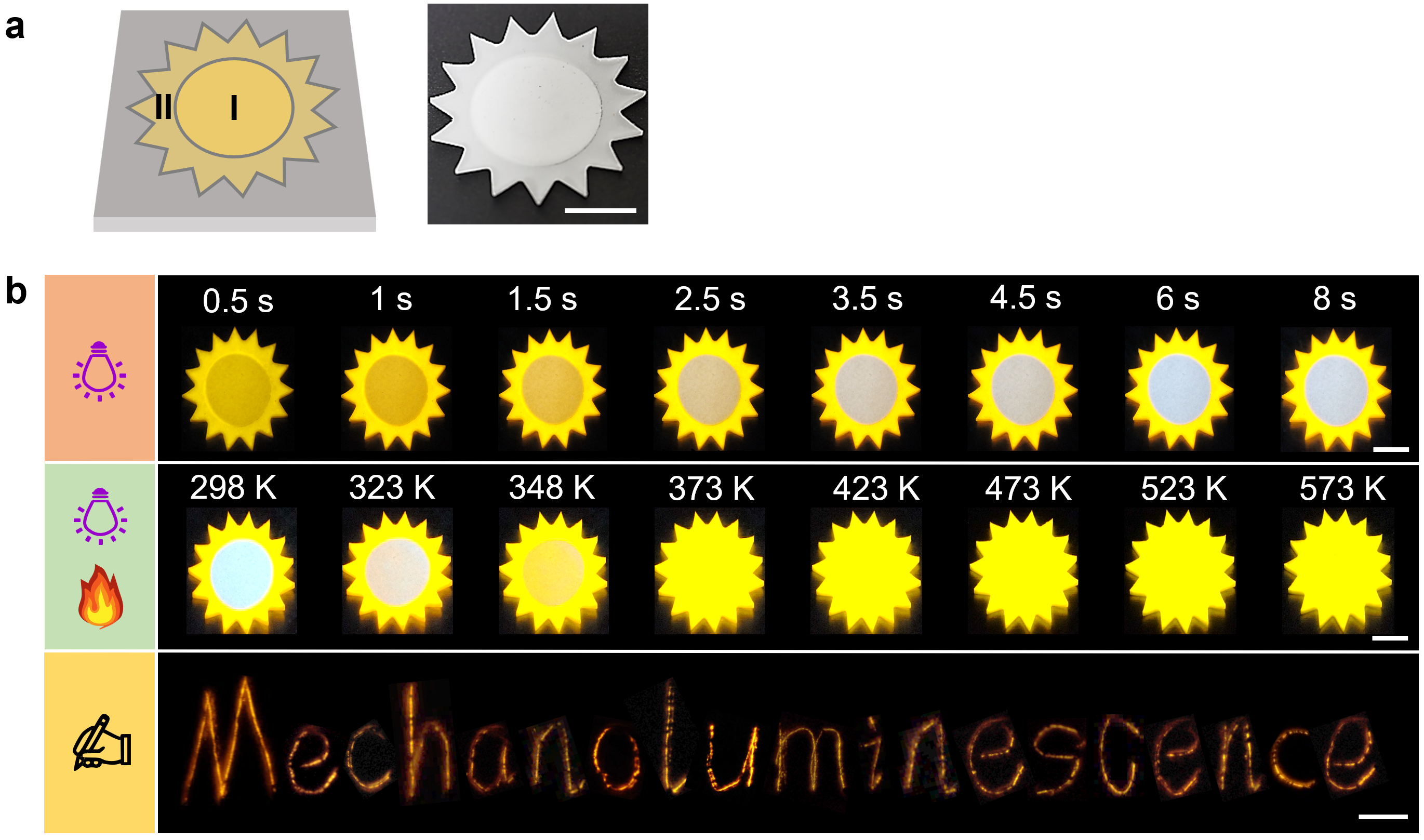


Supplementary Figure 37 | Multi-responsive multicolor luminescence using two CaGa_4_O_7_:Mn^2+^ phosphors for multidimensional dynamic displays. a, Schematic and optical photograph of the sun-shaped composite film made of phosphors and silicone rubber, where two phosphors (I: CaGa_4_O_7_:0.01 mol%Mn^2+^; II: CaGa_4_O_7_:0.1 mol%Mn^2+^) were embedded in Region I and Region II, respectively. b, Top: Time-dependent PL patterns excited by 254 nm light. The luminescent color of Region I changes from yellow to cool white within ten seconds, while the yellow emission in Region II remains unchanged. Middle: Temperature-dependent PL patterns. As the temperature increases, the luminescent color of Region I reverses from cool white to yellow, and the yellow emission remains intensely bright thereafter. In contrast, the yellow emission of Region II has no color change. Finally, both regions appear as a single entity to the naked eye, with the same luminescent color. Bottom: “Mechanoluminescence” pattern triggered by handwriting in Region I. Scale bars = 10 mm.

**Supplementary references**

1. Liu, P. et al. Trap depth engineering in MgGa_2_O_4_:Bi^3+^ for multicolor dynamic anti-counterfeiting, encryption and optical temperature sensing applications. *Chem. Eng. J.* **437,** 135389 (2022).
2. Hu, Y. et al. Thermal-responsive multicolor emission of single NaGdF_4_:Yb/Ce/Ho upconversion nanocrystals for anticounterfeiting application. *Nanophotonics* **9,** 2879 (2020).
3. Fan, Y. et al. Multimode dynamic photoluminescent anticounterfeiting and encryption based on a dynamic photoluminescent material. *Chem. Eng. J.* **393,** 124799 (2020).
4. Li, J.A. et al. Transient and persistent room-temperature mechanoluminescence from a white-light emitting AIEgen with tricolor emission switching triggered by light. *Angew. Chem. Int. Ed.* **57,** 6449–6453 (2018).
5. Wang, M. et al. Advanced luminescence anticounterfeiting based on dynamic photoluminescence and non-pre-irradiation mechanoluminescence. *Inorg. Chem.* **61,** 2911–2919 (2022).
6. Piotrowski, W.M. et al. Positive luminescence thermal coefficient of Mn^2+^ ions for highly sensitive luminescence thermometry. *Chem. Eng. J.* **464,** 142492 (2023).
7. Xu, H. et al. 2D perovskite Mn^2+^-doped Cs_2_CdBr_2_Cl_2_ scintillator for low-dose high-resolution X-ray imaging. *Adv. Mater.* **35,** 2300136 (2023).
8. Liu, Z. et al. Luminescence of native defects in MgGa_2_O_4_. *J. Electrochem. Soc.* **156,** H43−H46 (2009).
9. Jiang, B. et al. A self-activated MgGa_2_O_4_ for persistent luminescence phosphor. *J. Appl. Phys.* **124,** 063101 (2018).
10. Zheng, W. et al. Crystal field modulation-control, bandgap engineering and shallow/deep traps tailoring-guided design of a color-tunable long-persistent phosphor (Ca,Sr)Ga_4_O_7_:Mn^2+^,Bi^3+^. *Dalton Trans.* **48,** 253–265 (2019).
11. The Materials Project. 2020. "Materials Data on CaGa_4_O_7_ by Materials Project". United States. DOI:10.17188/1192773. https://www.osti.gov/servlets/purl/1192773. Pub date: Wed Jul 15 00:00:00 EDT 2020.
12. Zhang, J.C. et al. Eu^2+^/Eu^3+^-emission-ratio-tunable CaZr(PO_4_)_2_:Eu phosphors synthesized in air atmosphere for potential white light-emitting deep UV LEDs. *J. Mater. Chem. C* **2,** 312–318 (2014).
13. Pires, A.M. & Davolos, M.R. Luminescence of europium(III) and manganese(II) in barium and zinc orthosilicate. *Chem. Mater.* **13,** 21–27 (2001).
14. Meng, H. et al. Highly emissive and stable five-coordinated manganese(II) complex for X-ray imaging. *Laser Photonics Rev.* **15,** 2100309 (2021).
15. Zhang, J.C. et al. Discovering and dissecting mechanically excited luminescence of Mn^2+^ activators via matrix microstructure evolution. *Adv. Funct. Mater.* **31,** 2100221 (2021).
16. Kortüm, G., Braun, W., Herzog, D.C.G. Principles and techniques of diffuse-reflectance spectroscopy. *Angew. Chem. Int. Ed.* **2,** 333–341 (1963).
17. Pankove JI. Optical Processes in Semiconductors, Dover Publications, New York, 1971.
18. Liu, D. t al. Standardized measurement of dielectric materials’ intrinsic triboelectric charge density through the suppression of air breakdown. *Nat. Commun.* **13,** 6019 (2022).
19. Suo, H. et al. Rational design of ratiometric luminescence thermometry based on thermally coupled levels for bioapplications. *Laser Photonics Rev.* **30,** 1803388 (2020).
