# Supplementary material for: Dynamic multicolor emissions of multimodal phosphors by Mn2+ trace doping in self-activated CaGa4O7
Source: Nat Commun. 2024 Apr 13;15:3209. doi: 10.1038/s41467-024-47431-0 (PMC11016074; doi:10.1038/s41467-024-47431-0)
Supplement: Supplementary file 3 — Description of Additional Supplementary Files [file 41467_2024_47431_MOESM3_ESM.pdf]

Description of Additional Supplementary Files

File Name: Supplementary Movie 1
Description: Time-dependent dynamic multicolor photoluminescence

File Name: Supplementary Movie 2
Description: Temperature-dependent multicolor photoluminescence (10x speed).

File Name: Supplementary Movie 3
Description: Mechanoluminescence triggered by handwriting.
